# Supplementary material for: Selective Autophagy of Mitochondria on a Ubiquitin-Endoplasmic-Reticulum Platform
Source: Dev Cell. 2019 Sep 9;50(5):627–643.e5. doi: 10.1016/j.devcel.2019.06.016 (PMC6739445; doi:10.1016/j.devcel.2019.06.016)
Supplement: Document S1. Figures S1–S7 [file mmc1.pdf]

Developmental Cell, Volume *50*

Supplemental Information

Selective Autophagy of Mitochondria

on a Ubiquitin-Endoplasmic-Reticulum Platform

Maria Zachari, Sigurdur R. Gudmundsson, Ziyue Li, Maria Manifava, Ronak Shah, Matthew Smith, James Stronge, Eleftherios Karanasios, Caterina Piunti, Chieko Kishi-Itakura, Helena Vihinen, Eija Jokitalo, Jun-Lin Guan, Folma Buss, Andrew M. Smith, Simon A. Walker, Eeva-Liisa Eskelinen, and Nicholas T. Ktistakis

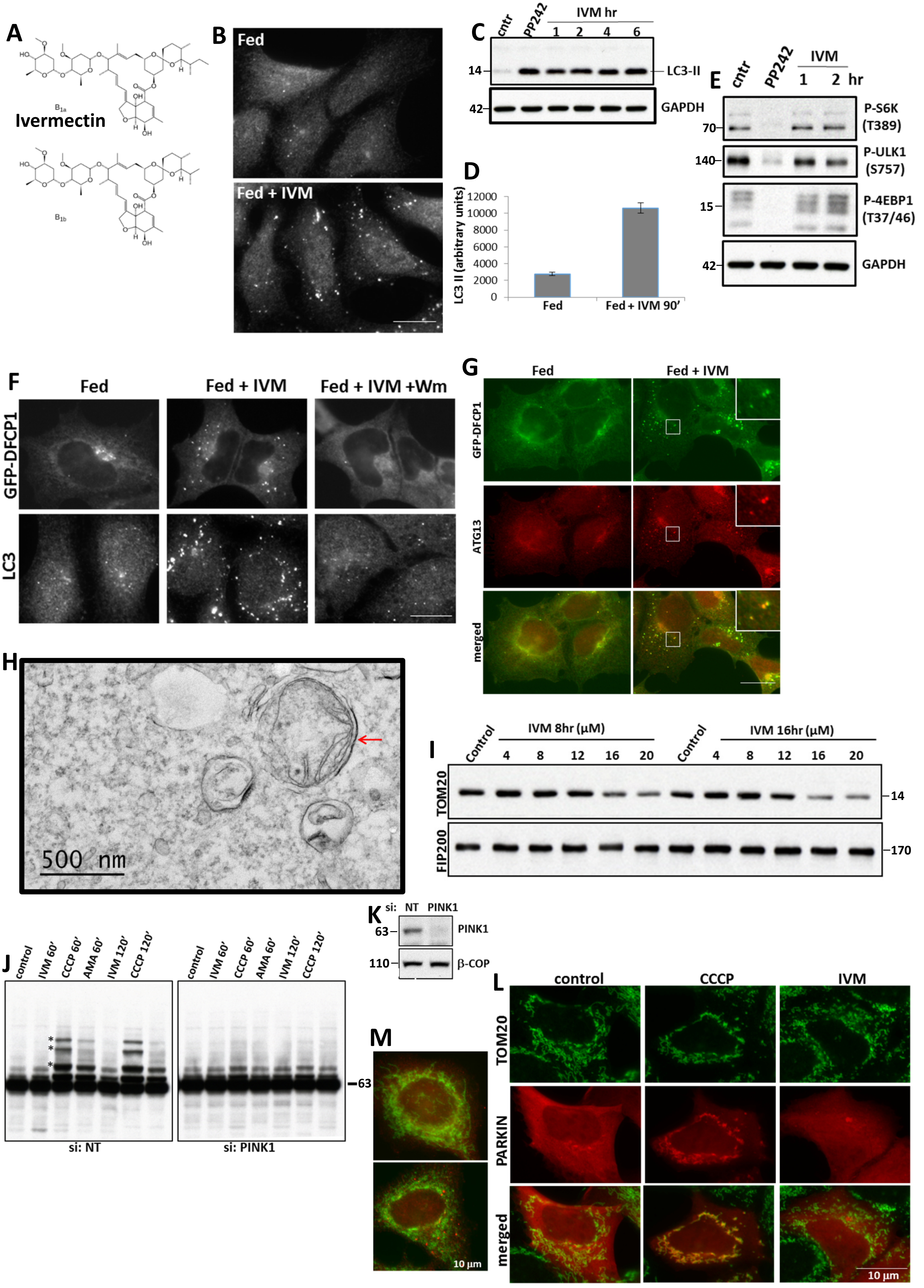

A
Ivermectin
B1a
B1b
B
Fed
Fed + IVM
C
cntr
PP242
IVM hr
1
2
4
6
14
LC3-II
42
GAPDH
D
LC3 II (arbitrary units)
Fed
Fed + IVM 90'
E
cntr
PP242
IVM
1
2 hr
P-S6K (T389)
70
P-ULK1 (S757)
140
P-4EBP1 (T37/46)
15
42
GAPDH
F
Fed
Fed + IVM
Fed + IVM +Wm
GFP-DFCP1
LC3
G
Fed
Fed + IVM
GFP-DFCP1
ATG13
merged
H
500 nm
I
Control
IVM 8hr (μM)
IVM 16hr (μM)
4
8
12
16
20
TOM20
FIP200
14
170
J
control
IVM 60'
CCCP 60'
AMA 60'
IVM 120'
CCCP 120'
63
si: NT
si: PINK1
K
si:
NT
PINK1
63
PINK1
110
β-COP
L
control
CCCP
IVM
TOM20
PARKIN
merged
10 μm
M
10 μm

Figure S1 related to Figure 1

IVM induces mitophagy. (A) Structure of IVM. (B-D)) HEKcells were treated with 15 μM IVM for 90 min, and examined for LC3 response by iffl and blots. (E)) HEKcells treated with PP242 for 1 hr or with IVM for 1 and 2 hr were lysed and immunoblotted for phospho-S6K, phopsho-ULK1 or phospho-4EBP1. (F) HEKcells stably expressing GFP-DFCP1 (top panels) or parental HEKcells (bottom panels) were treated with 15 μM IVM +/- 100 nM wortmannin and examined for DFCP1 or LC3. (G) HEKcells stably expressing GFP-DFCP1 were treated with 20 μM IVM for 90 min and immunolabelled for ATG13. (H) HEKcells treated with 15 μM IVM were fixed for EM examination. Arrow marks phagophore engulfing a single mitochondrion. (I) HEKcells were treated with the indicated amounts of IVM for 8 hr or 16 hr and then immunoblotted for TOM20 and FIP200 . (J-K)HEKcells were transfected with siRNAs against PINK1 or with non-targeting control (NT) and treated with IVM, CCCP or antimycin A before immunoblotting for MITOFUSIN 2 (J) or PINK1 (K). Asterisks indicate higher mobility forms of MITOFUSIN 2. (L) HEKcells were transiently transfected with mCherry-Parkin and treated for 90 min with CCCP or with IVM as indicated beforeTOM20 immunolabellimg (M) HeLa cells were treated with IVM for 90 min before immunolabelling with WIPI2 and TOM20.

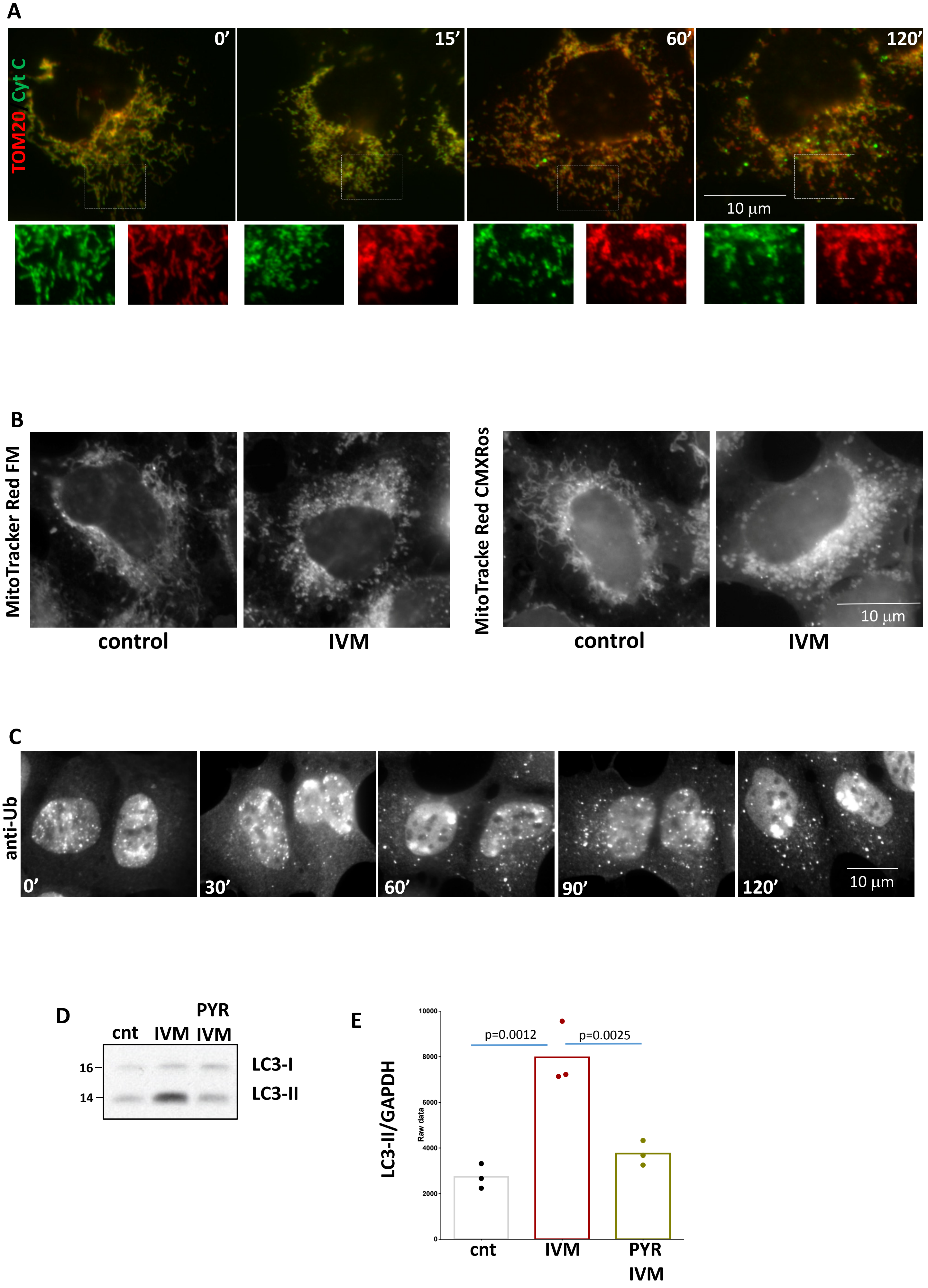
A
TOM20 Cyt C
0’
15’
60’
120’
10 µm
B
MitoTracker Red FM
control
IVM
MitoTracke Red CMXRos
control
IVM
10 µm
C
anti-Ub
0’
30’
60’
90’
120’
10 µm
D
cnt
IVM
PYR IVM
16
14
LC3-I
LC3-II
E
p=0.0012
p=0.0025
LC3-II/GAPDH
Raw data
10000
8000
6000
4000
2000
0
cnt
IVM
PYR IVM

Figure S2 related to Figure 1.

IVM does not induce mitochondrial permeabilization; IVM—induced mitophagy depends on ubiquitination. (A) HEK-293 cells were treated with 15 [illegible]M IVM for the times indicated and immunolabelled for TOM20 and cytochrome C as indicated. (B) HEK-293 cells were incubated with 50 ng/ml of Mitotracker Red FM or Mitotracker Red CMXRos as indicated for 10 min followed by addition of IVM for an additional 60 min. (C) Cells treated as in A were immunolabelled with antibodies to ubiquitin. (D) HEK-293 cells were treated with IVM in the presence or absence of PYR-41 and lysates were immunoblotted for LC3. (E) Triplicate experiment as shown in D is quantitated.

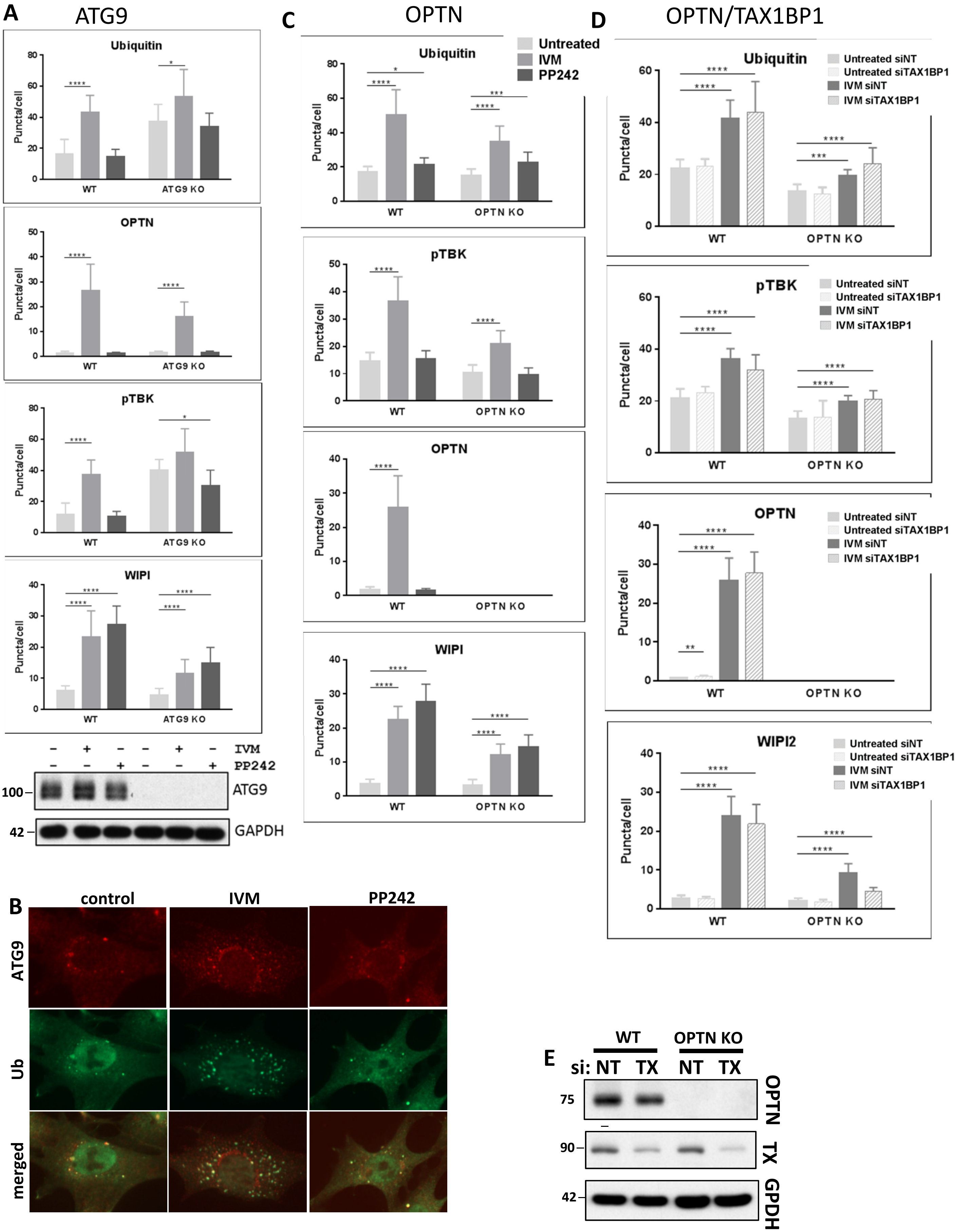
A
ATG9
Ubiquitin
Puncta/cell
WT
ATG9 KO
OPTN
pTBK
WIPI
IVM
PP242
100
42
ATG9
GAPDH
B
control
IVM
PP242
ATG9
Ub
merged
C
OPTN
Untreated
IVM
PP242
Ubiquitin
OPTN KO
pTBK
OPTN
WIPI
D
OPTN/TAX1BP1
Ubiquitin
Untreated siNT
Untreated siTAX1BP1
IVM siNT
IVM siTAX1BP1
pTBK
OPTN
WIPI2
E
WT
OPTN KO
si: NT TX NT TX
75
90
42
OPTN
TX
GPDH

Figure S3 related to Figures 2-3

Requirement for ATG9, Optineurin and Tax1bp in IVM-induced mitophagy. (A) Wild type MEF cells or ATG9 KOs were treated for 90 min with 15 mM IVM or with 1 mM PP242 and immunolabelled for ubiquitin, phospho-TBK1, Optineurin and WIPI2. Number of puncta for each condition are shown in the graphs. (B) MEF cells deleted for FIP200 were treated for 90 min with 15 mM IVM or with 1 mM PP242 and immunolabelled for ubiquitin and ATG9. (C) Wild type MEF cells or optineurin KOs were treated for 90 min with 15 μM IVM or with 1 μM PP242 and immunolabelled for ubiquitin, phospho-TBK1, Optineurin and WIPI2. Number of puncta for each condition is shown in the graphs. (D) Wild type and Optineurin KO MEFs as in A were transfected with siRNAs against Tax1bp or with a non-targeting oligonucleotide for 72 hrs. The cells were then treated with 15 μM IVM and were immunolabelled with ubiquitin, phospho-TBK1, optineurin and WIPI2. The number of puncta for each condition is shown in the graphs. (E) Lysates from wild type or Optineurin KO MEFs transfected with siRNAs to Tax1bp or with a non-targeting control (NT) and immunoblotted for the indicated proteins.

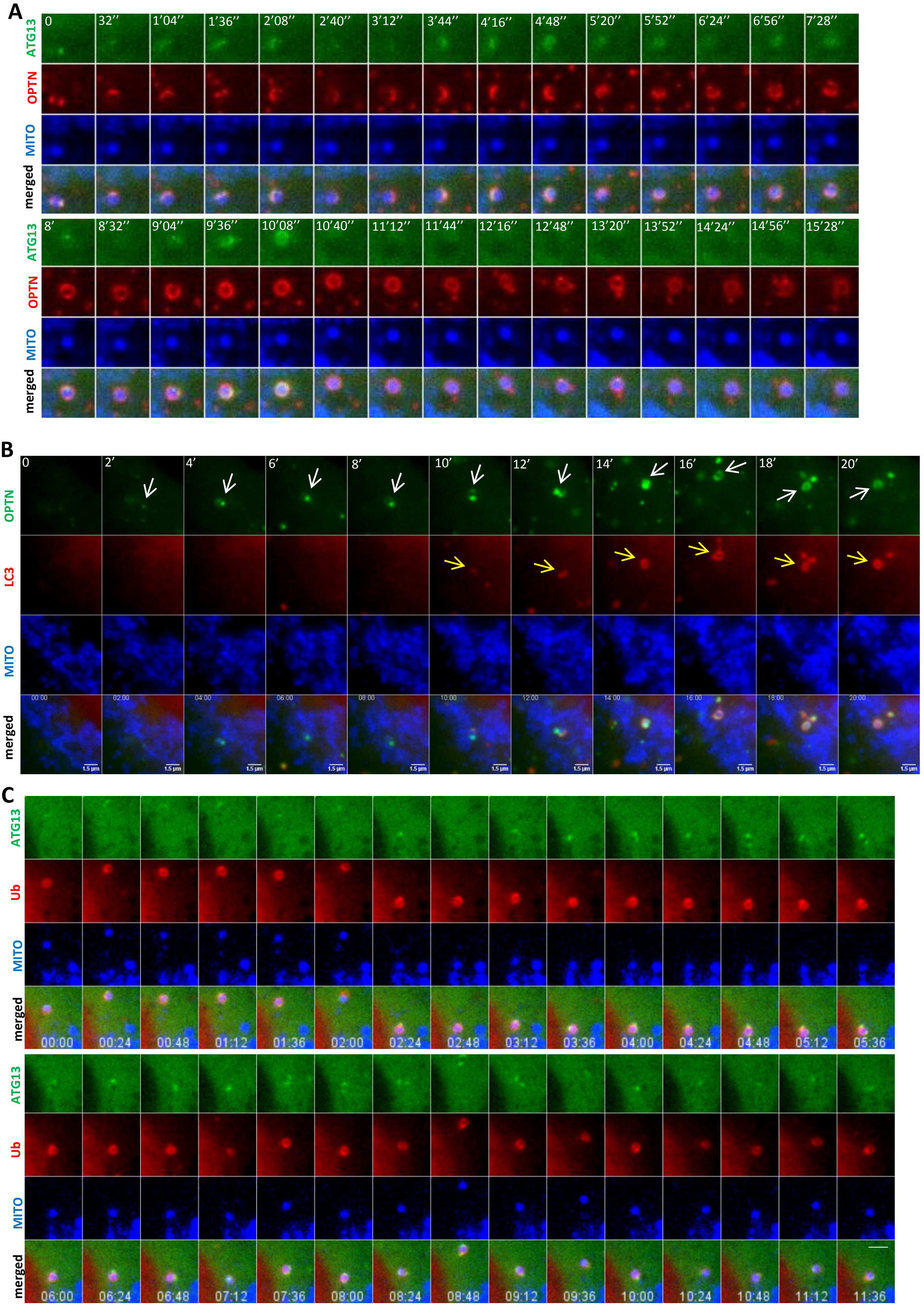
A
ATG13
OPTN
MITO
merged
0
32''
1'04''
1'36''
2'08''
2'40''
3'12''
3'44''
4'16''
4'48''
5'20''
5'52''
6'24''
6'56''
7'28''
8'
8'32''
9'04''
9'36''
10'08''
10'40''
11'12''
11'44''
12'16''
12'48''
13'20''
13'52''
14'24''
14'56''
15'28''
B
OPTN
LC3
MITO
merged
0
2'
4'
6'
8'
10'
12'
14'
16'
18'
20'
1.5 µm
C
ATG13
Ub
MITO
merged
00:00
00:24
00:48
01:12
01:36
02:00
02:24
02:48
03:12
03:36
04:00
04:24
04:48
05:12
05:36
06:00
06:24
06:48
07:12
07:36
08:00
08:24
08:48
09:12
09:36
10:00
10:24
10:48
11:12
11:36

Figure S4 related to Figure 4.

Dynamics of optineurin and ubiquitin during mitophagy. (A) Wide field live-imaging of HEK-293 cells stably expressing GFP-ATG13 and transiently transfected with mCherry-optineurin and CFP-MITO (A) or transiently transfected with GFP-optineurin, CFP-LC3 and mCherry-MITO (B) orstably expressing GFP-ATG13 and transiently transfected with mCherry-ubiquitin and CFP-MITO (C). IVM was added for 10 min and then cells were imaged for an additional 60 min. The bar represents 1 μm.

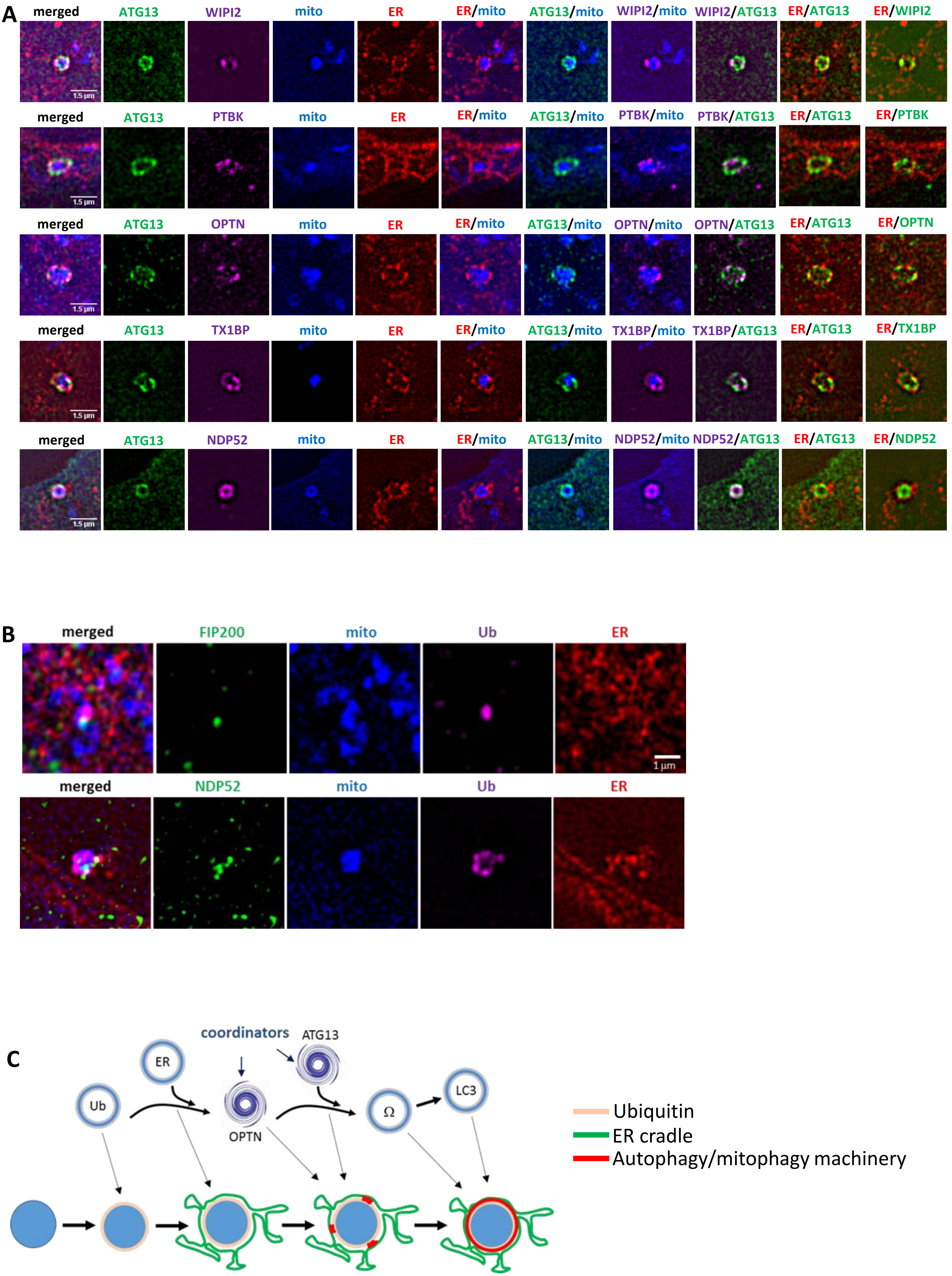

A
merged
ATG13
WIPI2
mito
ER
ER/mito
ATG13/mito
WIPI2/mito
WIPI2/ATG13
ER/ATG13
ER/WIPI2
1.5 µm
merged
ATG13
PTBK
mito
ER
ER/mito
ATG13/mito
PTBK/mito
PTBK/ATG13
ER/ATG13
ER/PTBK
1.5 µm
merged
ATG13
OPTN
mito
ER
ER/mito
ATG13/mito
OPTN/mito
OPTN/ATG13
ER/ATG13
ER/OPTN
1.5 µm
merged
ATG13
TX1BP
mito
ER
ER/mito
ATG13/mito
TX1BP/mito
TX1BP/ATG13
ER/ATG13
ER/TX1BP
1.5 µm
merged
ATG13
NDP52
mito
ER
ER/mito
ATG13/mito
NDP52/mito
NDP52/ATG13
ER/ATG13
ER/NDP52
1.5 µm
B
merged
FIP200
mito
Ub
ER
1 µm
merged
NDP52
mito
Ub
ER
C
coordinators
ATG13
ER
Ub
OPTN
Ω
LC3
Ubiquitin
ER cradle
Autophagy/mitophagy machinery

Figure S5 related to Figure 5.

(A) An expanded set of the images shown in Figure 8 D. Four-color SIM images of mitochondrial fragments (blue) during IVM-induced mitophagy and associated with ATG13 (green), optineurin (purple), WIPI2 (purple), phospho-TBK1 (purple) Tax1BP (purple), NDP-52 (purple) and ER (red) as indicated. Shown are the four individual channels, the four-color overlay and two-color overlays of the various components as indicated. (B) Four-color SIM images of mitochondrial fragments (blue) during IVM-induced mitophagy in HEK-293 cells lacking ATG13. Staining for ER (red) and other components is as indicated. (C) Dynamics of mitophagy as revealed in this work. Several components are involved in the engulfment of the mitochondrial target (top diagram): some cover it smoothly (ubiquitin, ER, omegasomes, LC3, continuous lines) whereas others appear to oscillate on and off and translocate to multiple sites on the target (optineurin and ATG13 shown as an example, swirling lines). We imagine that these components co-ordinate the translocation of the rest of the machinery, including the lipidation complex and LC3. One way to explain these dynamics is that the ubiquitination step leads to some stable interaction of the mitochondrial target with an ER cradle, which provides the spatial clues for organizing the translocation of the autophagy/mitophagy machinery (bottom diagram).

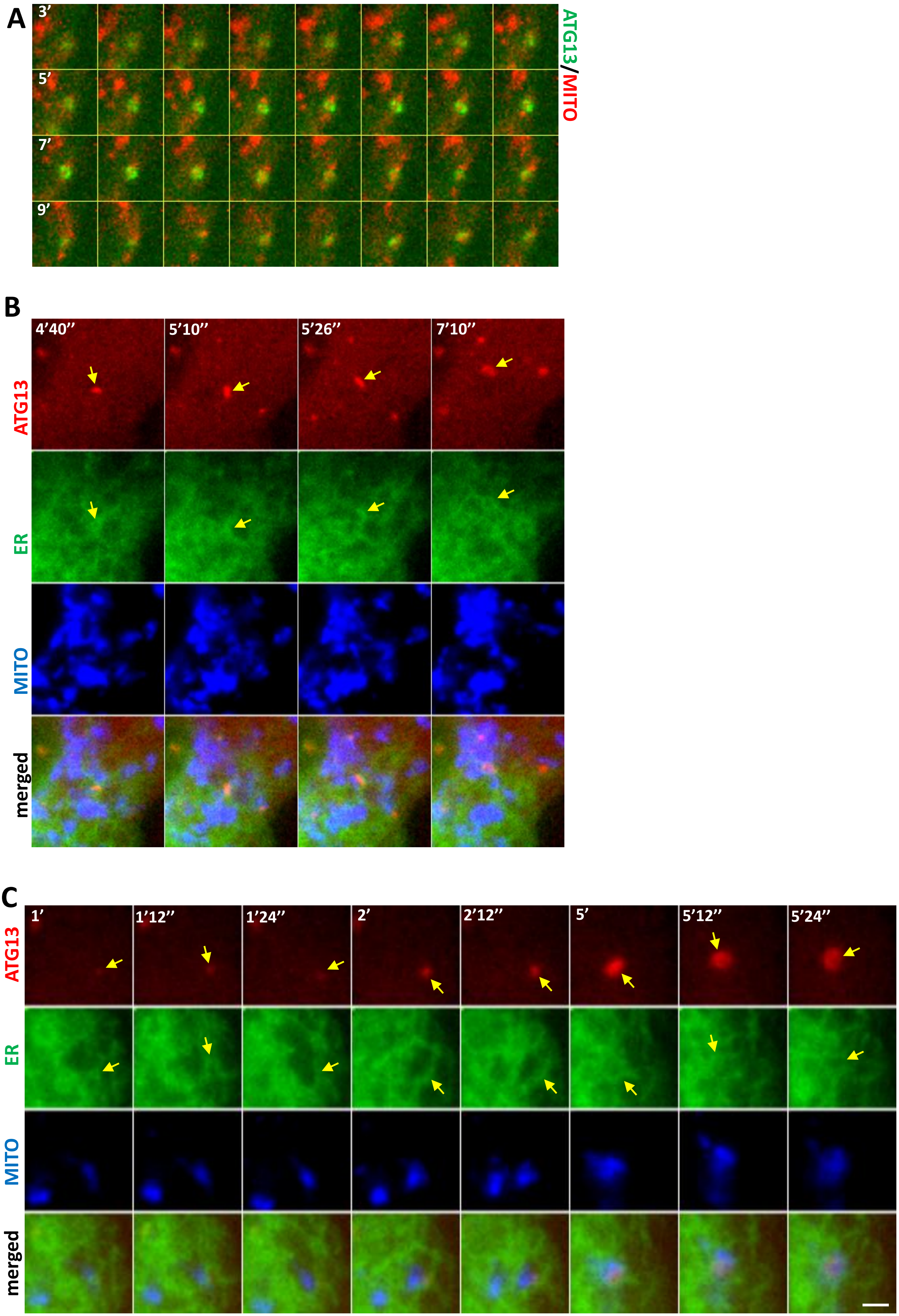

A
3’
5’
7’
9’
ATG13/MITO
B
4’40’’
5’10’’
5’26’’
7’10’’
ATG13
ER
MITO
merged
C
1’
1’12’’
1’24’’
2’
2’12’’
5’
5’12’’
5’24’’
ATG13
ER
MITO
merged

Figure S6 related to Figures 4-5.

Dynamics of mitophagy induced by Antimycin A/Oligomycin (A/O) treatment. (A) HEK-293 cells expressing GFP-ATG13 and mCherry-MITO were treated with A/O for 8 h and then imaged every 12 sec.. (B-C) HEK-293 cells expressing GFP-ATG13, mCherry-MITO and CFP-ER were treated with A/O for 8 h (C) or 18 (D) h and imaged every 12 sec as shown in the selected panels. Arrows indicate the position of ATG13 and ER structures during the mitochondrial engulfment process. The bar in all panels represents 1 μm.

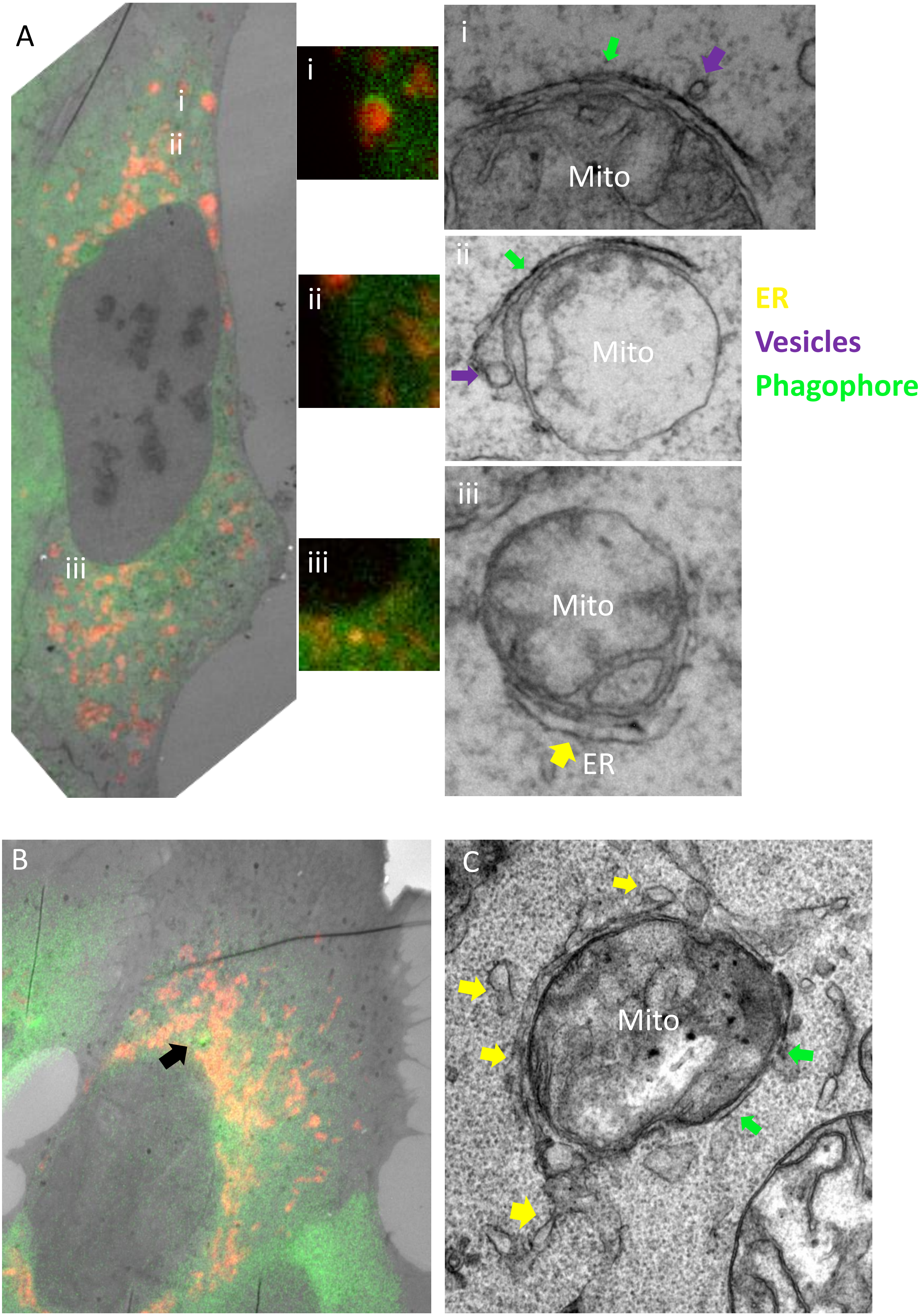
A
i
ii
iii
i
ii
iii
i
Mito
ii
Mito
iii
Mito
ER
ER
Vesicles
Phagophore
B
C
Mito

Figure S7 related to Figure 6.

Correlative light-electron microscopy images of IVM-induced mitophagy. (A) A cell with 3 simultaneous mitophagosomes (i, ii, iii). Events i and ii show a forming phagophore tightly next to the mitochondrion while event iii shows an ER-like structure next to the mitochondrion, although an ATG13 signal is evident in live imaging in all three events. The absence of a phagophore when ATG13 is seen to translocate to the mitochondrion is reminiscent of the event shown in Fig 9 M-P and probably represents the earliest intermediates in mitophagy pathway. (B) A mitophagy event showing how ER (yellow arrows) and phagophore (green arrows) wrap around the targeted mitochondrial fragment.
