# Supplementary material for: Selective Autophagy of Mitochondria on a Ubiquitin-Endoplasmic-Reticulum Platform
Source: Dev Cell. 2019 Sep 9;50(5):627–643.e5. doi: 10.1016/j.devcel.2019.06.016 (PMC6739445; doi:10.1016/j.devcel.2019.06.016)
Supplement: Document S2. Article plus Supplemental Information [file mmc9.pdf]

Developmental Cell

Article

Selective Autophagy of Mitochondria on a Ubiquitin-Endoplasmic-Reticulum Platform

Graphical Abstract

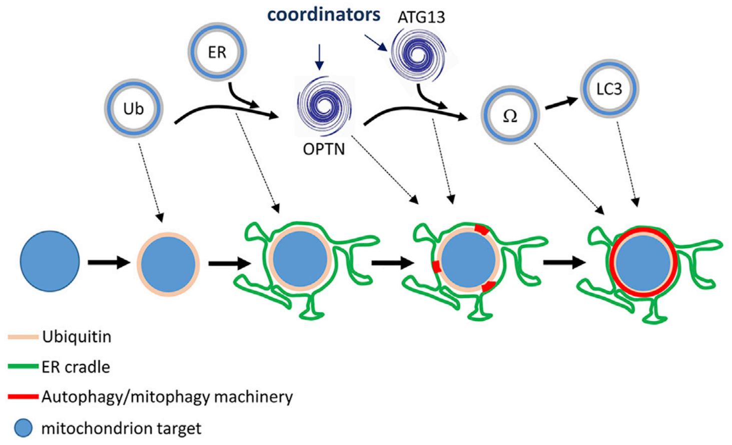

Authors

Maria Zachari,
Sigurdur R. Gudmundsson, Ziyue Li, ...,
Simon A. Walker, Eeva-Liisa Eskelinen,
Nicholas T. Ktistakis

Correspondence

nicholas.ktistakis@babraham.ac.uk

In Brief

Zachari et al. use genetic and imaging experiments to define the ordered steps of mitophagy induction in response to the drug ivermectin. Their findings suggest that damaged mitochondria are ubiquitinated and dynamically encased in ER strands that then provide the platform for the formation of mitophagosomes.

Highlights

- Mitophagy requires coordination of machineries for target recognition and engulfment
- IVM-induced mitophagy depends on TRAF2/CIAP1/CIAP2 for ubiquitination of mitochondria
- Ubiquitinated mitochondria within ER regions facilitate targeting and autophagy
- ULK complex is required, but not for initiation, with FIP200 acting before ATG13

Zachari et al., 2019, Developmental Cell *50*, 627–643
September 9, 2019 © 2019 The Authors. Published by Elsevier Inc.
https://doi.org/10.1016/j.devcel.2019.06.016

CellPress

Developmental Cell
Article

Cell Press

Selective Autophagy of Mitochondria on a Ubiquitin-Endoplasmic-Reticulum Platform

Maria Zachari,[1] Sigurdur R. Gudmundsson,[2,8] Ziyue Li,[1,8] Maria Manifava,[1,8] Ronak Shah,[1] Matthew Smith,[1] James Stronge,[1] Eleftherios Karanasios,[1] Caterina Piunti,[1] Chieko Kishi-Itakura,[3] Helena Vihinen,[4] Eija Jokitalo,[4] Jun-Lin Guan,[7] Folma Buss,[3] Andrew M. Smith,[5] Simon A. Walker,[1] Eeva-Liisa Eskelinen,[6,9] and Nicholas T. Ktistakis[1,10,*]
[1]Signalling Programme, Babraham Institute, Cambridge, UK
[2]Department of Biosciences, University of Helsinki, Helsingfors, Finland
[3]Cambridge Institute for Medical Research, University of Cambridge, Cambridge, UK
[4]Institute of Biotechnology, University of Helsinki, Helsingfors, Finland
[5]Division of Medicine, University College London, London, UK
[6]Molecular and Integrative Biosciences Research Programme, University of Helsinki, Helsingfors, Finland
[7]Department of Cancer Biology, University of Cincinnati College of Medicine, Cincinnati, OH, USA
[8]These authors contributed equally
[9]Present address: Institute of Biomedicine, University of Turku, Turku, Finland
[10]Lead Contact
*Correspondence: nicholas.ktistakis@babraham.ac.uk
https://doi.org/10.1016/j.devcel.2019.06.016

SUMMARY

The dynamics and coordination between autophagy machinery and selective receptors during mitophagy are unknown. Also unknown is whether mitophagy depends on pre-existing membranes or is triggered on the surface of damaged mitochondria. Using a ubiquitin-dependent mitophagy inducer, the lactone ivermectin, we have combined genetic and imaging experiments to address these questions. Ubiquitination of mitochondrial fragments is required the earliest, followed by auto-phosphorylation of TBK1. Next, early essential autophagy proteins FIP200 and ATG13 act at different steps, whereas ULK1 and ULK2 are dispensable. Receptors act temporally and mechanistically upstream of ATG13 but downstream of FIP200. The VPS34 complex functions at the omegasome step. ATG13 and optineurin target mitochondria in a discontinuous oscillatory way, suggesting multiple initiation events. Targeted ubiquitinated mitochondria are cradled by endoplasmic reticulum (ER) strands even without functional autophagy machinery and mitophagy adaptors. We propose that damaged mitochondria are ubiquitinated and dynamically encased in ER strands, providing platforms for formation of the mitophagosomes.

INTRODUCTION

Autophagy is a conserved pathway for nutrient supply during periods of starvation, classified as non-selective autophagy, or for degradation of intracellular large structures that are pathogenic or have become damaged, classified as selective autophagy (Mizushima and Komatsu, 2011; Mizushima et al., 2011; Ktistakis and Tooze, 2016). In both pathways, a novel double membrane organelle termed autophagosome is formed in the cytosol that then engulfs its cargo for eventual delivery to the lysosomes and degradation (Feng et al., 2014; Ohsumi, 2014). For non-selective autophagy, the cargo is total cytosol, and its degradation in the lysosomes generates nutrients essential during starvation (Dunlop and Tee, 2014; Mony et al., 2016). In contrast, specific elimination of large membrane structures—damaged mitochondria, endoplasmic reticulum (ER) fragments, or bacterial pathogens—is the purview of selective autophagy and constitutes an essential quality control system (Okamoto, 2014; Stolz et al., 2014; Randow and Youle, 2014; Anding and Baehrecke, 2017).

The pathway of autophagosome formation in response to starvation is now well understood, although the exact origin of the autophagosomal membrane is still a matter of debate (Lamb et al., 2013; Bento et al., 2016). For autophagosomes that originate from within PI3P-enriched regions of the ER termed omegasomes, the pathway starts by inactivation of the protein kinase complex mTORC1 and the concomitant activation of the autophagy-specific ULK protein kinase complex composed of the protein kinase ULK1 (or its homolog ULK2) and the adaptors FIP200, ATG13, and ATG101 (Saxton and Sabatini, 2017; Wong et al., 2013). Activated ULK complex translocates to tubulovesicular regions of the ER marked by ATG9 vesicles, and these sites attract the lipid kinase complex termed VPS34 complex I, which produces PI3P and forms omegasomes (Walker et al., 2008; Karanasios et al., 2016). PI3P within the omegasome membrane attracts members of the WIPI family of proteins that in turn bind to the protein ATG16 and mediate the covalent modification of the LC3 and GABARAP proteins with phosphatidylethanolamine, which is an important requirement for the formation of autophagosomes (Wilson et al., 2014; Yu et al., 2018).

The process of selective autophagy requires a set of proteins connecting the targeted cargo to the autophagic machinery and a signal on the cargo to mark it for sequestration (Johansen and Lamark, 2011; Rogov et al., 2014). Selective autophagy receptors are responsible for bridging cargo with the forming autophagosome. In yeast, they include Atg32 for mitochondrial autophagy (mitophagy, Okamoto et al., 2009; Kanki et al.,

This is an open access article under the CC BY license (http://creativecommons.org/licenses/by/4.0/).

CellPress

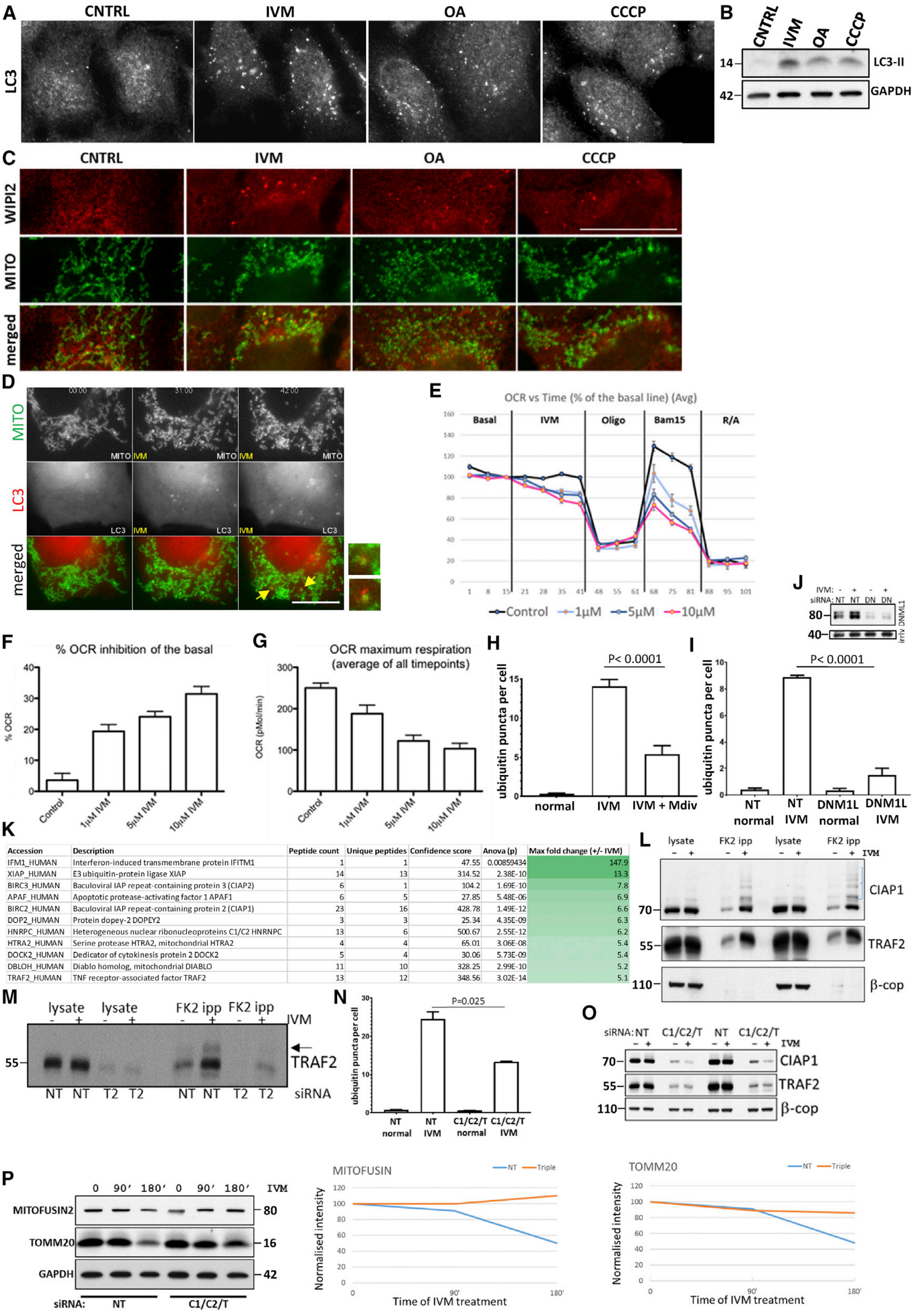

Accession	Description	Peptide count	Unique peptides	Confidence score	Anova (p)	Max fold change (+/- IVM)
IFM1_HUMAN	Interferon-induced transmembrane protein IFITM1	1	1	47.55	0.00859434	147.9
XIAP_HUMAN	E3 ubiquitin-protein ligase XIAP	14	13	314.52	2.38E-10	13.3
BIRC3_HUMAN	Baculoviral IAP repeat-containing protein 3 (CIAP2)	6	1	104.2	1.69E-10	7.8
APAF_HUMAN	Apoptotic protease-activating factor 1 APAF1	6	5	27.85	5.48E-06	6.9
BIRC2_HUMAN	Baculoviral IAP repeat-containing protein 2 (CIAP1)	23	16	428.78	1.49E-12	6.6
DOP2_HUMAN	Protein dopey-2 DOPEY2	3	3	25.34	4.35E-09	6.3
HNRPC_HUMAN	Heterogeneous nuclear ribonucleoproteins C1/C2 HNRNPC	13	6	500.67	2.55E-12	6.2
HTRA2_HUMAN	Serine protease HTRA2, mitochondrial HTRA2	4	4	65.01	3.06E-08	5.4
DOCK2_HUMAN	Dedicator of cytokinesis protein 2 DOCK2	5	4	30.06	5.73E-09	5.4
DBLOH_HUMAN	Diablo homolog, mitochondrial DIABLO	11	10	328.25	2.99E-10	5.2
TRAF2_HUMAN	TNF receptor-associated factor TRAF2	13	12	348.56	3.02E-14	5.1

Figure 1. Inducers of Mitophagy in Mammalian Cells and IVM Action

(A–C) HEK293 cells treated for 2 h with 15 μM IVM, for 8 h with 10 μM oligomycin and 10 μM antimycin A (OA), or for 8 h with 4 μM CCCP and stained for LC3 (A) or immunoblotted for LC3. (B) Cells treated as above, stained for TOMM20 (MITO) and WIPI2.

(legend continued on next page)

2009), Atg36 and Atg30 for autophagy of peroxisomes (pexophagy, Motley et al., 2012; Nazarko et al., 2014), Atg39 and Atg40 for autophagy of ER membranes (Mochida et al., 2015), and Atg19/Atg34 for the Cvt pathway (Scott et al., 2001; Suzuki et al., 2010; Watanabe et al., 2010). Equivalent and homologous proteins exist for mammals and for many types of cargo (Khaminets et al., 2016). Receptors interact with the autophagic machinery via LC3 and GABARAP-interacting regions that bridge autophagosomal membranes with targeted cargo, and such a simple bi-valent interaction could, in principle, enable engulfment (Birgisdottir et al., 2013). However, the autophagic machinery must also be involved in this process since it is responsible for generating lipidated LC3 and GABARAP residing on autophagosomal membranes. The current work aims to identify the dynamics and hierarchical coordination between the autophagic machinery and the selective autophagy receptors.

The pathway of mitophagy has been extensively studied since it was first described (Lemasters, 2005). The "eat me" signals on damaged mitochondria initiating this process (Randow and Youle, 2014) can be divided into ubiquitin-dependent and ubiquitin-independent (Khaminets et al., 2016; Yamano et al., 2016). The former rely on ubiquitination of mitochondrial outer membrane proteins in response to damage that is then recognized by mitophagy receptors for recruitment of the LC3 and GABARAP proteins (Dikic, 2017; Kwon and Ciechanover, 2017). A paradigm is the mitophagy pathway regulated by the PINK1 and Parkin proteins (Narendra et al., 2008; Nguyen et al., 2016) where several receptors such as optineurin, NDP-52, Tax1BP1, and p62 translocate to damaged mitochondria (Lazarou et al., 2015). Mitophagy "eat me" signals independent of ubiquitination rely on specific mitochondrial proteins acting as mitophagy receptors (Khaminets et al., 2016; Roberts et al., 2016).

Although mitophagy plays an essential role for mitochondrial homeostasis *in vivo*, the exact signals that trigger it at the organismal level are still relatively obscure (Whitworth and Pallanck, 2017; Rodger et al., 2018). In contrast, at least 14 different pharmacological agents induce mitophagy in tissue culture cells (Georgakopoulos et al., 2017). Taking advantage of our experimental models previously used to follow the dynamics of non-selective autophagy in mammalian cells, we have now examined the dynamics of mitophagy including the origin of the membrane used for mitophagy and the coordination between autophagy and mitophagy machineries during the engulfment step.

RESULTS

IVM Characterization

We searched for mitophagy inducers not requiring overexpression of Parkin (or of any other protein) so as to avoid extremely strong, potentially non-physiological activation of this pathway. The protonophore CCCP (Narendra et al., 2008) and, more recently, a combination of oligomycin and antimycin A (OA, Lazarou et al., 2015) induce canonical PINK1-Parkin-dependent mitophagy upon Parkin overexpression. In mouse embryonic fibroblasts (MEFs) with undetectable expression of Parkin, we did not observe mitophagy with these compounds (data not shown). In contrast, HEK293 cells with moderate endogenous Parkin expression showed a mitophagy response after 8 h of treatment. Another compound that induced this pathway within 2 h of treatment was the heterocyclic lactone ivermectin (IVM). In all three cases, we observed translocation of LC3 to autophagy puncta (Figure 1A) and detected formation of LC3 type II—a lipidated form of LC3 diagnostic of autophagy induction—by immunoblots (Figure 1B). In addition, early autophagy components, such as WIPI2, working downstream of the PI3P-dependent step (Polson et al., 2010), translocated to puncta and co-localized with mitochondrial fragments (Figure 1C). To verify that these puncta were mitochondria targeted by the autophagy machinery we used live imaging (Figure 1D; Video S1). Upon IVM treatment, mitochondria fragmented within 10 min and LC3 puncta that appeared 15 min later associated with these fragments (Figure 1D arrows). The other two compounds also induced similar dynamics (see later sections for OA treatment) but less frequently. Of note, IVM induced this response within 30 min of treatment, which provides a convenient tool for live-imaging studies.

We carried out additional experiments to characterize IVM, which is a well-known anti-parasitic compound (Campbell et al., 1984; Crump and Ōmura, 2011), in the mitophagy pathway (Figure S1). HEK293 cells treated with IVM showed translocation of LC3 to punctate structures by immunofluorescence (Figure S1B) and formation of LC3 type II comparable to autophagy induction with the mTOR inhibitor PP242 (Figures S1C and S1D). The IVM-induced response did not depend on mTOR inactivation (Figure S1E) but was sensitive to the VPS34 inhibitor wortmannin (Figure S1F) and used an omegasome intermediate (Figure S1G). Mitophagy induced by IVM showed the hallmarks of the response by electron microscopy (EM) (formation of

(D) Live-cell imaging of HEK293 cells expressing CFP-LC3 and mCherry-MITO and treated with 15 μM IVM. Shown are selected time points; arrows mark mitochondrial fragments targeted by LC3. See Video S1 for the whole sequence. Scale bar, 10 μm.

(E–G) OCR of HEK293 cells treated with IVM. Time course and percent inhibition are plotted as shown.

(H) HEK293 cells treated with 15 μM IVM and 40 μm mdiv-1 as indicated for 45 min, stained for ubiquitin, and puncta per cell determined. Means of two experiments done in duplicate are shown.

(I and J) HEK293 cells treated with siRNA against DNM1L or with a non-targeting (NT) control for 72 h. After incubation with 15 μM IVM, cells were stained for ubiquitin and puncta per cell were determined. Means of two experiments done in duplicate are shown.

(K) HEK-293 cells untreated or treated with 15 μM IVM for 45 min, lysed, and immunoprecipitated with ubiquitin antibodies. Samples were analyzed by mass spectrometry and the top 11 hits enriched after IVM treatment are shown.

(L) Samples as in (K) were blotted for CIAP1, TRAF2, or β-COP (a loading control).

(M) HEK293 cells treated with siRNA against TRAF2 or NT control for 72 h were treated as in (K) and immunoblotted for TRAF2.

(N) HEK-293 cells treated with siRNA against CIAP1, CIAP2 and TRAF2 or with NT control for 96 h. After treatment with 15 μM IVM and staining for ubiquitin, puncta per cell were determined. Means of three experiments done in duplicate are shown.

(O) Parallel samples were lysed and blotted for CIAP1, TRAF2 or β-COP.

(P) Cells downregulated for CIAP1, CIAP2, and TRAF2 as in (N) and (O) above were incubated with IVM and the levels of mitochondrial proteins TOMM20 and MITOFUSIN 2 were determined by immunoblots and quantitated.

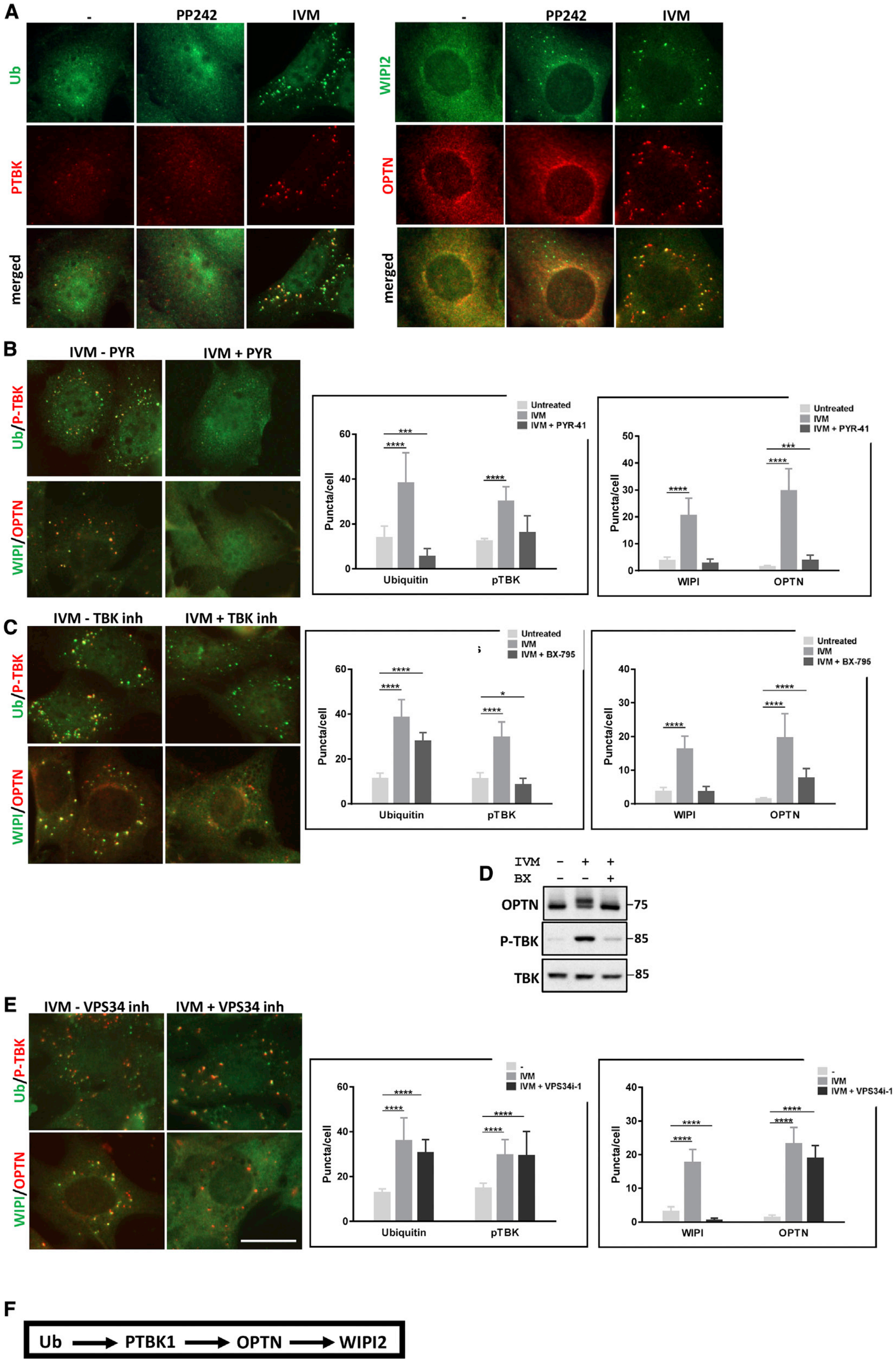

Figure 2. Response of Mitophagy Components to IVM in MEFs and Hierarchy of Steps

(A) MEFs treated for 90 min with 15 μM IVM or with 1 μM PP242 and immunolabeled for ubiquitin and P-TBK1 (left panels) or WIPI2 and optineurin (right panels).

(B) MEFs treated for 90 min with 15 μM IVM and 30 μM PYR-41 and immunostained as above. Puncta were quantitated (graphs to the right).

(legend continued on next page)

mitophagosomes surrounding mitochondria, Figure S1H) and caused significant degradation of mitochondrial proteins (Figure S1I). However, this mitophagy response did not require PINK1 and Parkin (Figures S1J–S1M). In further characterization, we showed that IVM did not cause extensive mitochondrial permeabilization (Figures S2A and S2B) but enhanced cytosolic ubiquitin puncta (Figure S2C). Of note, inhibition of ubiquitination with the broad inhibitor PYR-41 (Yang et al., 2007), reduced the effects of IVM on LC3 type II formation (Figures S2D and S2E).

Because treatment with IVM causes fragmentation of mitochondria and induces ubiquitination, we determined if its mechanism of action was similar to other known mitophagy inducers such as OA that modulate mitochondrial respiration during mitophagy. Indeed, oxygen consumption rate (OCR) was significantly reduced by IVM at the concentration range used in our assays (Figures 1E–1G). We also determined whether fragmentation of mitochondria upon IVM addition was a prerequisite for their ubiquitination by using either a specific chemical inhibitor of the DNM1L GTPase (Mdiv) involved in mitochondrial fission or siRNA against the DNM1L enzyme. Both inhibited ubiquitination (Figures 1I and 1J). Thus, IVM causes ubiquitin-dependent mitophagy without relying on the PINK1 and Parkin ubiquitination system. To identify candidate target proteins and the ubiquitin E3 ligases involved, we isolated and identified ubiquitinated proteins enriched in IVM-treated cells versus untreated cells (Figure 1K). Of the top 11 proteins differentially enriched by mass spectrometry, we verified that CIAP1 (also known as BIRC2) and TRAF2 were substantially enriched on anti-ubiquitin columns upon IVM treatment (Figures 1L and 1M), and slower migrating forms of CIAP1 (Figure 1L, blue bracket) or TRAF2 (Figure 1M, arrow) were enriched in those columns. Although we did not find specific antibodies for CIAP2, we assume that it is also present in the columns based on its interaction with CIAP1 and the proteomics data. To examine if CIAP1, TRAF2, and CIAP2 were functionally involved in IVM action, we downregulated all three by siRNA and measured IVM-induced ubiquitin puncta in cells (Figures 1N and 1O) and IVM-induced degradation of TOMM20 and MITOFUSIN 2 (Figure 1P). Reduction of the three proteins inhibited ubiquitination and degradation by 50%, indicating that the three proteins are at least partially involved in IVM action. Of note, both TRAF2 and CIAP1 have recently been implicated in autophagy and mitophagy responses (Meng et al., 2010; Tang et al., 2013; Hu et al., 2017) with TRAF2 functioning downstream of the drug celastrol for ubiquitination and mitophagy (Hu et al., 2017).

Genetic Studies

To delineate the mechanism of mitophagy, we used inhibitors and MEFs deleted in various autophagy and mitophagy genes. A set of antibodies to endogenous reporter proteins allowed differentiation of mitophagy induction from non-selective autophagy (Figure 2A). These were anti-ubiquitin, anti-phospho(S172)-TBK1 (P-TBK1, a kinase involved in selective autophagy [Wild et al., 2011; Heo et al., 2015]), anti-optineurin (a mitophagy adaptor), and anti-WIPI2. Untreated cells showed a diffuse signal with all four antibodies. Cells induced for autophagy with PP242 showed a punctate signal for WIPI2 but diffuse signal for the other three reporters. In contrast, IVM treatment caused the translocation of all four reporters to punctate signals that co-localized with each other (Figure 2A). Thus, WIPI2 puncta that do not correspond to optineurin puncta are likely to be specific for non-selective-autophagy, whereas P-TBK1 and ubiquitin puncta formed when optineurin and WIPI2 puncta also appear, are likely to be mitophagy specific. To ensure that IVM-induced puncta were related to mitophagy, we stained these cells with antibodies to a mitochondrial protein and either P-TBK1 or WIPI2. These puncta co-localized with mitochondrial fragments (data not shown). With those markers, we then determined the hierarchical relationship between ubiquitination (using the PYR-41 inhibitor), TBK1 auto-phosphorylation (using the BX-795 inhibitor [Feldman et al., 2005]) and VPS34 activation (using the VPS34-IN1 inhibitor [Bago et al., 2014; Dowdle et al., 2014]). PYR-41 inhibited translocation of all four reporters to puncta, indicating that ubiquitination is the most upstream (Figure 2B). Inhibition of TBK1 phosphorylation reduced puncta of P-TBK1, WIPI2, and optineurin, but not of ubiquitin, indicating that TBK1 activation is downstream of ubiquitination but upstream of activation of the receptors and of the autophagy machinery at the VPS34 step (Figure 2C). Of note, BX-795 inhibited IVM-induced phosphorylation of TBK1 and the IVM-dependent mobility shift of optineurin (Figure 2D). Finally, inhibition of VPS34 did not affect translocation of ubiquitin, P-TBK1, and optineurin to IVM-induced puncta but inhibited translocation of WIPI2 to puncta (Figure 2E). From these data, we delineated an initial sequence of steps for mitophagy (shown in Figure 2F).

We then used MEFs deleted in early autophagy genes to delineate this pathway with respect to the autophagic machinery. In the absence of FIP200, a component of the ULK complex, translocation of ubiquitin and phospho-TBK1 was evident, but that of optineurin and WIPI2 was inhibited (Figure 3A, left column). In the absence of ATG13, another component of the ULK complex, ubiquitin, P-TBK1, and optineurin translocated to punctate structures upon IVM treatment, but WIPI2 did not (Figure 3A, middle column). Unexpectedly, the absence of both ULK1 and ULK2 (the kinases of the ULK complex) did not inhibit translocation of any of the four components, indicating that early steps of mitophagy could proceed normally (Figure 3A, right column). For all experiments, we included samples treated with PP242. This was important in all cases but especially for the ULK1/ULK2 knockout (KO) samples where we could show that non-selective autophagy (induced by PP242) was still dependent on those two kinases, unlike mitophagy. An unanticipated conclusion from these results was that IVM-induced mitophagy exhibited a differential requirement for two of the ULK complex proteins with FIP200 acting before ATG13. With respect to FIP200, we

(C) Cells analyzed as in (B) but treated with 15 μM IVM and 5 μM BX-795.

(D) Cells treated with IVM alone or with IVM and BX-795 were immunoblotted for optineurin, P-TBK1, and TBK1.

(E) Cells analyzed as in (B) but treated with 15 mM IVM and 4 μM VPS34-IN1.

Scale bar, 15 μm.

(F) Hierarchy of translocation of the 4 autophagy/mitophagy components (ubiquitin, P-TBK1, optineurin, and WIPI2).

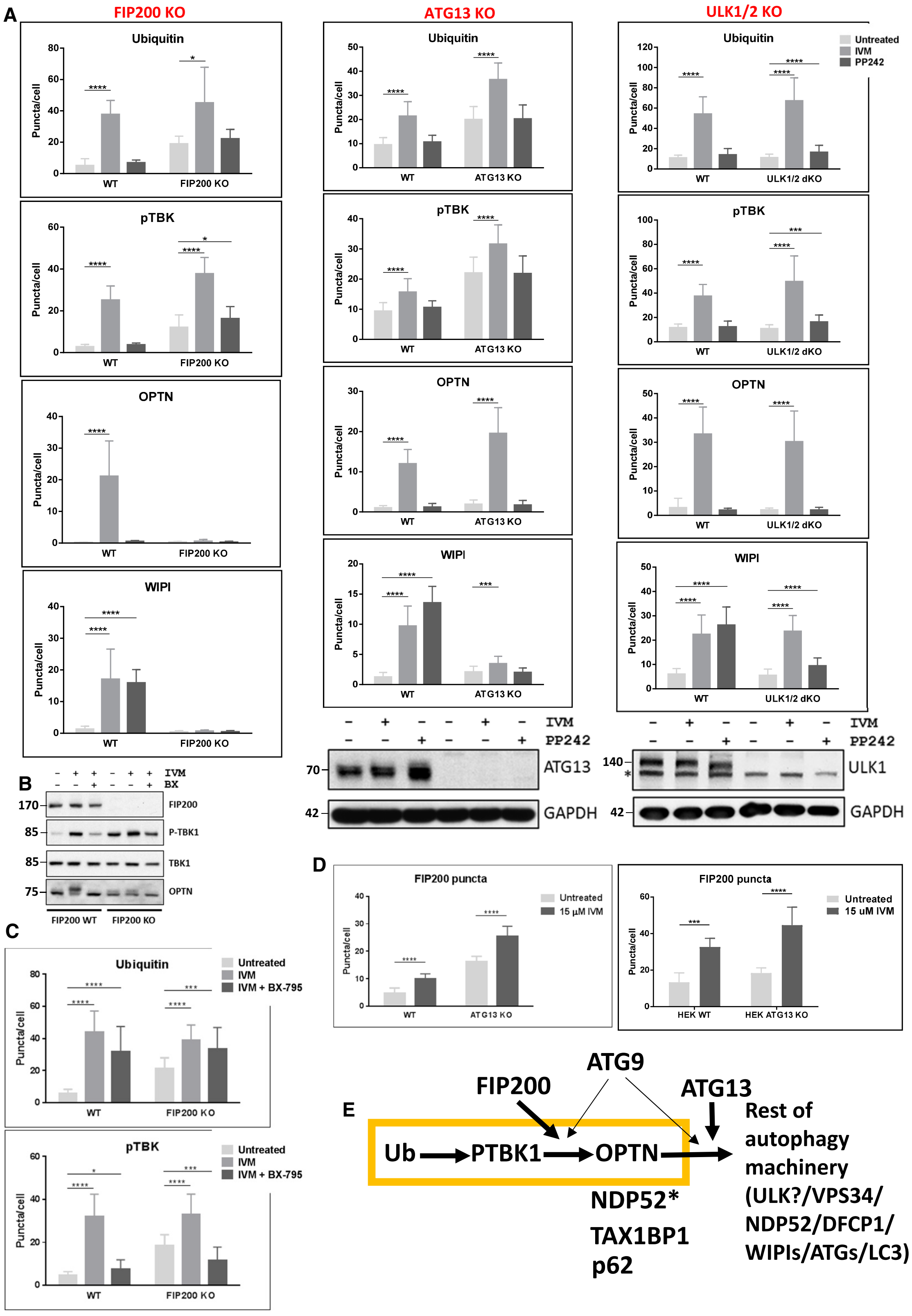

Figure 3. Requirement of Autophagy Proteins FIP200, ATG13, and ULK1/2 in IVM-Induced Mitophagy

(A) Wild-type MEFs or matched FIP200 KO (left column graphs), ATG13 KO (middle column graphs), or ULK1/2 KO (right column graphs) treated for 90 min with 15 µM IVM or with 1 µM PP242 and immunolabeled for ubiquitin, P-TBK1, optineurin, and WIPI2. The number of puncta for each condition is shown in the graphs. Blots show absence of the relevant protein (for FIP200 KO MEFs see C).

(legend continued on next page)

showed, in addition, that FIP200 KO cells showed elevated amounts of P-TBK1 by immunoblots whereas IVM treatment still increased those levels further (Figure 3B). Formation of the phosphorylated, lower mobility form of optineurin in the FIP200 KO cells was significantly suppressed (Figure 3B), in agreement with the immunofluorescence results (Figure 3A, left column) that indicate that optineurin puncta do not form in response to IVM treatment. Although the FIP200 KO cells showed elevated levels of P-TBK1 puncta, this was still sensitive to the BX-795 inhibitor indicating that this basal elevated state was reversible (Figure 3C). Our data implied that without ATG13, FIP200 could still translocate to puncta in response to IVM, and this was the case for both ATG13 KO MEFs and HEK293 cells carrying a deletion of the ATG13 gene (Figure 3D). Analysis of the IVM response in ATG9 KO cells showed translocation of all four proteins but both optineurin and WIPI2 puncta were half of the levels of the corresponding wild-type cells (Figure S3A), indicating that ATG9 has an important but not essential role. To better explore if ATG9 can target mitophagy structures separately from the rest of the autophagy machinery we examined its localization upon IVM treatment in FIP200 KO cells. In these cells, which are severely inhibited for mitophagy and only the ubiquitination and TBK1 activation steps are evident, ATG9 was still able to target ubiquitin-enriched mitophagy puncta (Figure S3B).

Based on the MEF data, we derived the preliminary pathway shown in Figure 3E for the early steps of mitophagy. Core steps targeting mitochondria are ubiquitination, auto-phosphorylation of TBK1, and mobilization of receptors such as optineurin, in this order (Figure 3E, components in orange box). The early autophagy machinery engages with this sequence at 3 stages: FIP200 is essential downstream of TBK1 activation, ATG13 works downstream of receptor activation, whereas ATG9 is not essential but enhances the response at the FIP200 and ATG13 steps. The rest of the autophagy machinery, including activated VPS34 and the PI3P effectors WIPI2 and DFCP1, translocates to mitophagy sites downstream of the ATG13 step. One prediction of this model is that autophagy proteins involved in lipidation will all function downstream of this core sequence, and we found this to be the case for ATG5, ATG3, and ATG7 KO MEFs (data not shown).

For PINK1-Parkin-dependent mitophagy, only optineurin and NDP-52 among the receptors are essential for engulfment and delivery (Lazarou et al., 2015). MEFs express a homolog of NDP-52, non-functional for selective autophagy, so we examined if IVM-dependent mitophagy was reliant on optineurin alone. In MEFs deleted for optineurin, the response to IVM was suppressed but not eliminated (Figure S3C). In cells both carrying a deletion for optineurin and treated with Tax1BP1 siRNA, IVM-induced translocation of WIPI2 was significantly inhibited (Figures S3D and S3E). Of note, those cells still showed a response of WIPI2 to PP242, suggesting that the reduced response to IVM was specific for mitophagy and not an inhibition of general non-selective autophagy. We concluded from these results that mitophagy in MEFs requires the presence of optineurin and Tax1BP1, with the former having a more important role.

Dynamics of Mitophagy

We next examined the dynamics of the mitophagy response using a combination of live imaging, super resolution, and EM and related this to the genetic and pharmacological analysis above. To assess engulfment, we imaged cells expressing the omegasome reporter DFCP1, the autophagy protein LC3, and a mitochondrial marker. Complete engulfment of mitochondria fragments by LC3 was seen in those cells, accompanied by translocation of DFCP1 and dynamic interaction with LC3 during the process (Figure 4A; Video S2A). Of note, whereas LC3 engulfment was smooth and continuous, the engagement of omegasomes with mitochondria was more intricate with frequent changes of direction and a discontinuous rate. Instances were also noted where multiple omegasomes formed on the mitochondria accompanied by multiple LC3-positive structures, before all coalesced into a single structure that engulfed its target (Figure 4B; Video S2B). The ATG13 protein—as part of the ULK complex—translocated to the forming mitophagy structures not smoothly but discontinuously, appearing to oscillate on and off as it moved around the targeted mitochondria (Figure 4C, green channel, and Video S3A). Importantly, the LC3 structure that surrounded the same mitochondrial fragment did so smoothly, initiating from one region of the spherical mitochondrial piece and going around it (Figure 4C, red channel). These videos were filmed at one frame every 10 s, and it is possible that the ATG13 movements were too fast to be captured accurately. When we captured frames every 1s (Figure 4D; Video S3B), we saw that ATG13 structures targeted the mitochondrial fragments in multiple waves, with each wave lasting 30–60 s and initiating from different regions of the fragments. Thus, the discontinuous ATG13 dynamics of the slower video in panel C are likely due to this oscillatory behavior. To estimate the frequency of these oscillatory recruitments, we counted all events where we could distinguish an ATG13 signal partially (or fully) surrounding a mitochondrial fragment at some point during the time course. In this way, we excluded events showing an interaction between ATG13 and a mitochondrial membrane without the former surrounding the latter that could be due to nucleation of an autophagosome for non-selective autophagy near a mitochondrion. Under those conditions, 100% of events implicated in mitophagy showed oscillatory behavior of ATG13. These data have been used to derive a model for this early step in the pathway (Dalle Pezze et al., personal communiation).

The initial dynamics of optineurin translocation to forming mitophagosomes resembled those of ATG13 in being discontinuous

(B) Wild-type or FIP200 KO MEFs were treated with 15 μM IVM and 5 μM BX-795 as indicated before lysis and immunoblotting for FIP200, P-TBK1, TBK1, and optineurin as indicated.

(C) Wild-type and FIP200 KO MEFs treated with 15 μM IVM and 5 μM BX-795, and immunolabeled for ubiquitin or P-TBK1. Number of puncta is shown in the graphs.

(D) ATG13 KO MEFs (left graph) or ATG13 KO HEK293 cells (right graph) were treated with 15 μM IVM and immunolabeled for FIP200. The number of puncta is shown.

(E) Hierarchical involvement of mitophagy and autophagy components after IVM addition.

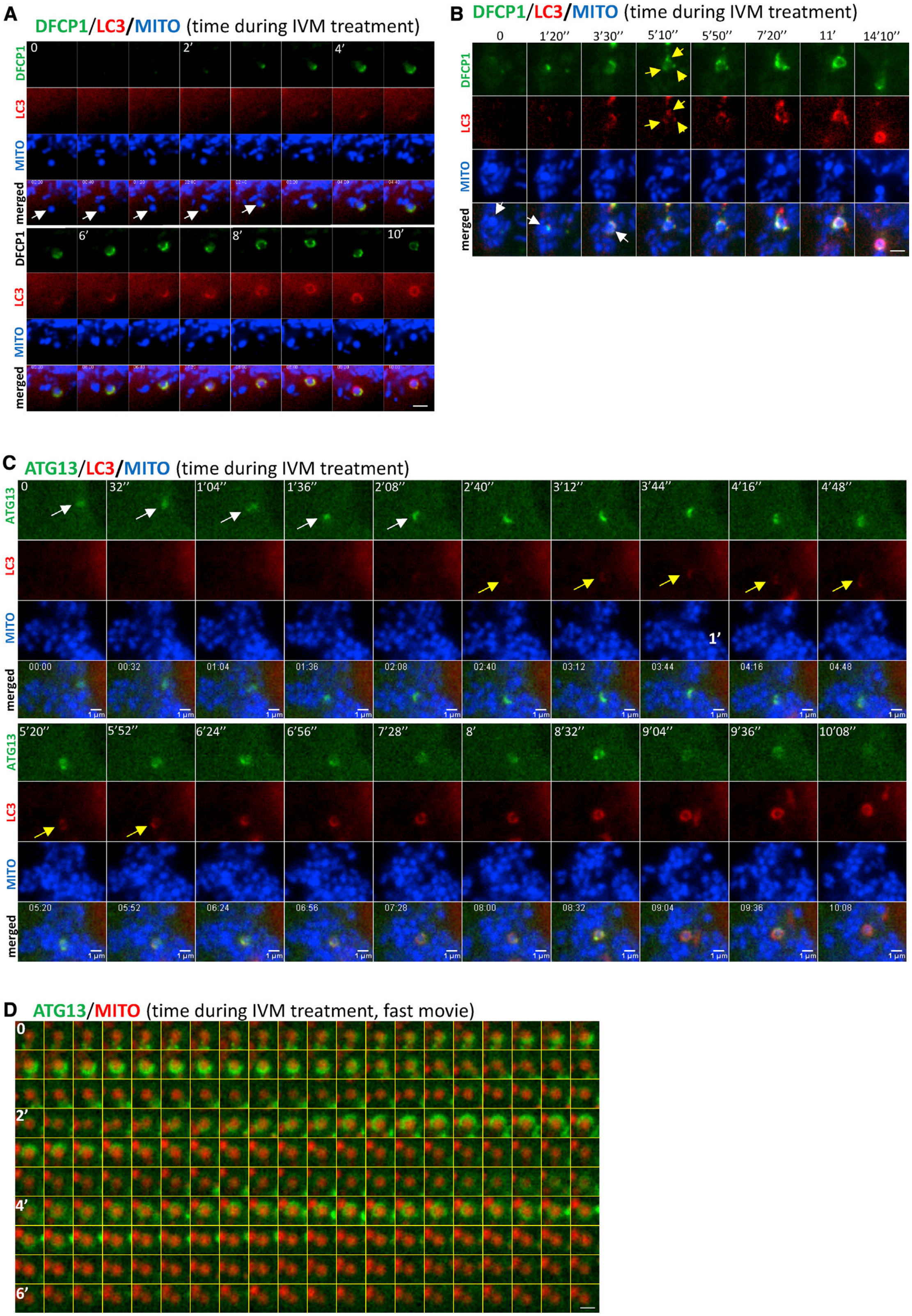

Figure 4. Dynamics of Omegasomes and ATG13 during Mitophagy

(A) Live-cell imaging of HEK293 cells expressing GFP-DFCP1, CFP-LC3, and mCherry-MITO, treated with IVM. A mitochondrial fragment is engulfed by DFCP1 and LC3 (white arrows). Whole sequence in Video S2A.

(legend continued on next page)

and jerky, although eventually the optineurin structures completely engulfed the mitochondrial fragments and stayed associated with them (Figure S4A; Video S4A). Interestingly, the initial movements of optineurin around the targeted structures were not synchronous and did not coincide spatially with ATG13 (Figure S4A), though both ATG13 and optineurin first engaged with their targets synchronously. In contrast, engagement of optineurin with its target preceded the engagement of LC3 by almost 10 min (Figure S4B; Video S4B). The temporal order of engagement as revealed by these videos (first optineurin, then ATG13, then DFCP1 and LC3) is consistent with the hierarchical scheme in Figure 3D. Given that the whole process initiates with a ubiquitinated mitochondrial fragment, we then used live imaging to verify that the ATG13 jerky movements around the targeted mitochondria were on structures fully outlined by ubiquitin (Figure S4C; Video S5).

The Role of the ER

For non-selective autophagy via omegasome intermediates, the ER provides a cradle for assembly of autophagosomal membranes. For selective autophagy, the role of the ER is unknown. We observed that ATG13-enriched autophagosomal structures engulfing mitochondrial fragments showed a strong coincidence with the underlying ER (Figure 5A; Video S6) for long periods during engulfment that was maintained as the targeted mitochondria moved around the cell. When mitochondria were surrounded by the ER, the ATG13 dynamics were always seen in association with the strands (Figure 5B, short sequence leading to the engulfed structure marked with arrows, and Figure 5C). Close apposition of targeted mitochondria to ER domains was also seen by super-resolution microscopy. SIM imaging of cells treated with IVM and stained for ATG13, WIPI2 (or, instead, P-TBK1 or optineurin), mitochondria, and ER showed that the engulfed mitochondrial fragments were encased within ER strands where the autophagy and mitophagy machinery was also assembled (Figures 5D and S5A). To address the possibility that such structures were formed only when mitophagy proceeded to completion, we used HEK293 cells lacking ATG13. In these cells, FIP200 and ubiquitin still respond to IVM (see for example Figure 3D) but downstream events are blocked (see Figure 3E). SIM analysis of those cells showed that FIP200 and NDP52 were still capable of translocating to ubiquitin-marked mitochondrial fragments associating with ER (Figure S5B), although these structures did not appear fully formed. We obtained similar results with all of the KO lines that responded either partially or fully to IVM: the formed structures were associated with fragmented mitochondria.

Examples of tight association between the ER and forming mitophagosomes are evident in some previous publications using Parkin overexpression (see Figure 2A in Yoshii et al., 2011), suggesting a generalized ER involvement for mitophagy. To explore this further, we used live imaging after OA treatment (shown to induce canonical PINK1-Parkin-dependent mitophagy, Lazarou et al., 2015, and first introduced in Figure 1). Under this alternative mitophagy induction, we showed that ATG13 structures translocated and rotated on mitochondrial fragments akin to the IVM response (Figure S6A). Importantly, translocation of ATG13 to forming mitophagosomes was on ER regions and the full engulfment by ATG13 was on fragments encased by the ER (Figures 6B and 6C, note regions marked by arrows). These results suggest that the basic characteristics of the mitophagy pathway we have described are maintained across more than one induction protocol.

To examine the spatial relationship between mitophagosomes and the ER at higher resolution, we used a combination of live microscopy followed by EM (Figure 6) first described for our autophagy work (Walker et al., 2017). Cells expressing ATG13 and a mitochondrial marker were treated with IVM during live imaging, fixed on stage, and prepared for EM serial sectioning or tomography. We found examples where ATG13 structures surrounding mitochondria (Figures 6B–6D and S7) were recognized after EM preparation in the form of double membrane phagophores (Figures 6A, 6E–6G, and S7). The double membrane phagophore surrounded the mitochondrion very tightly (Figures 6A and 6E–6G). In this particular example, three separate phagophores are evident (green in Figures 6E–6G), and interestingly, the space devoid of phagophores was tightly occupied by a membrane cistern resembling ER (yellow in Figures 6E–6G). A reconstruction of this event shows a very tight association between the ER, the phagophore, and the targeted mitochondrion (Figures 6H–6L; Video S7). Additional examples of the engulfment process are shown in Figure S7. In addition to proximity to the ER and phagophore (especially Figures S7B and S7C), other vesicles can be seen in the surrounding region, and in one example, a mitochondrion is targeted by ER strands without the double-membrane phagophore being visible (Figure 7Aiii). This is a particularly informative event because the ATG13-positive structure was very early based on live imaging, and it is likely that a double-membrane phagophore had not yet formed. Another such example is shown in Figures 6M–6P in more detail. The ATG13 particle formed around the mitochondrial fragment was clearly identified in the live imaging, but no phagophore was apparent in the EM tomogram. Instead, the targeted mitochondrion was surrounded by ER strands and a few vesicles. We hypothesize that this may be one of the earliest visible intermediates in the engulfment process where elements of the early autophagy machinery, such as proteins of the ULK complex and ATG9 vesicles, associate with the targeted mitochondrion and with the ER before a proper phagophore begins to form.

With respect to the ubiquitination step, super-resolution microscopy revealed a close association of the ubiquitin signal with the targeted mitochondria and with both autophagy and mitophagy components (Figure 7A, top two rows). The ubiquitin layer on the targeted mitochondria was also in tight apposition to the ER (Figure 7A, last three rows). The temporal relationship

(B) Cells as in (A) but with a larger mitochondrial fragment (white arrows) targeted by 3 separate DFCP1- and LC3-positive phagophores (yellow arrows). Whole sequence in Video S2B. Scale bar, 2 μm.

(C) Imaging of HEK293 cells expressing GFP-ATG13, CFP-LC3, and mCherry-MITO, treated with IVM. Engagement of an ATG13 structure with a mitochondrial fragment (white arrows) followed by LC3 (yellow arrows). Whole sequence in Video S3A.

(D) HEK293 cells expressing GFP-ATG13 and mCherry-MITO, treated with IVM. Oscillation of ATG13 around a single mitochondrial fragment. Whole sequence in Video S3B. Scale bar, 1 μm.

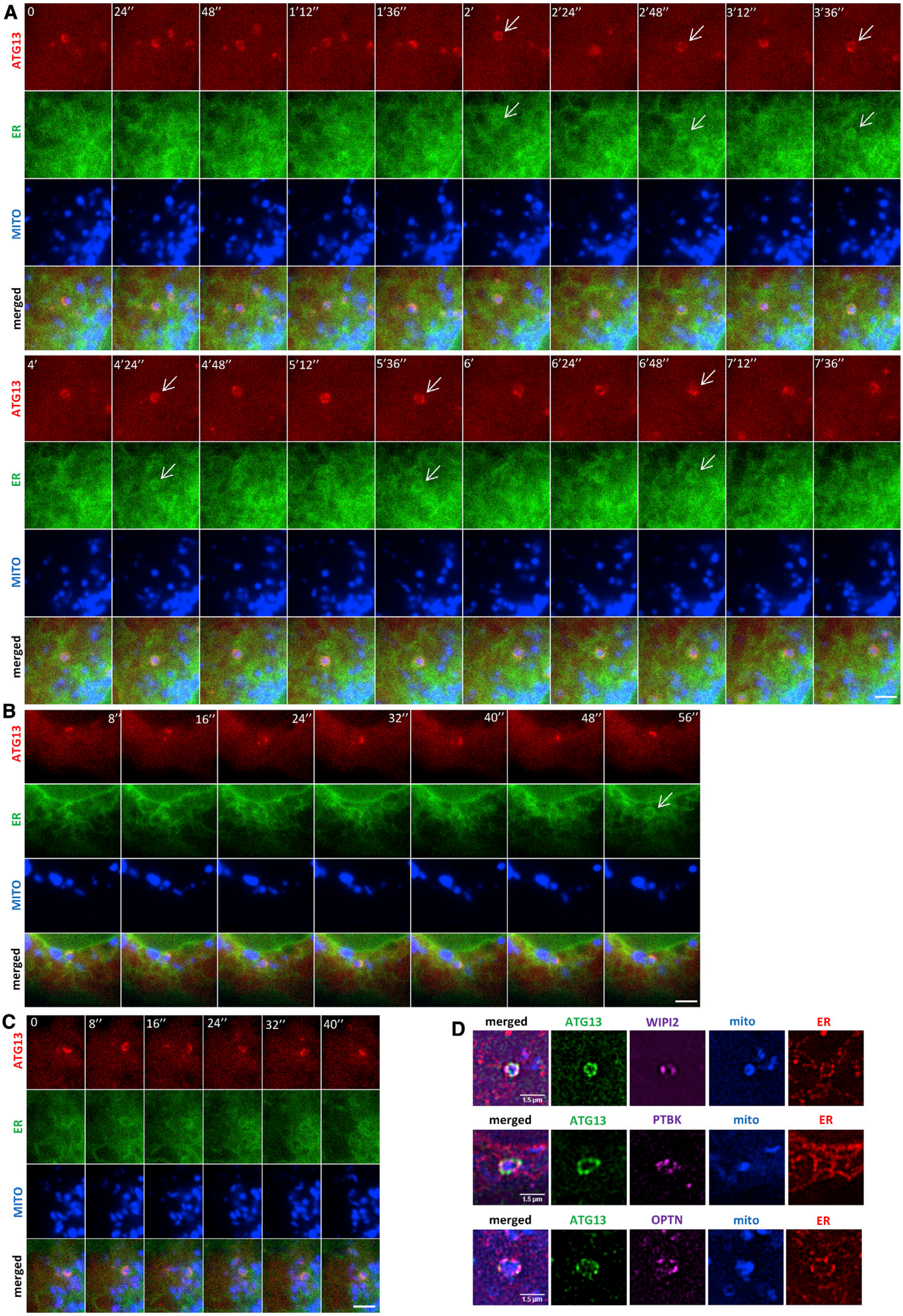

Figure 5. The ER during Mitophagy

(A) Imaging of HEK293 cells expressing GFP-ATG13, CFP-ER, and mCherry-MITO, treated with IVM. Arrows point to instances where ATG13 and ER coincide. Whole sequence in Video S6.

(legend continued on next page)

of the ubiquitinated mitochondrial fragments with the ER was complicated. When fragments first became ubiquitinated (with instantaneous timing and without discernible intermediate stages of engulfment), they interacted with ER strands but were not encased by them (Figure 7B). Several minutes later, the ubiquitin and the ER signal overlapped significantly, with the ER surrounding the ubiquitinated structures (Figure 7B, white arrows in the last two frames). Once such an overlap was established, it lasted for over 10 min, and it was evident as the mitochondrial fragments moved around the cell (Figure 7C, white arrows). Association of ubiquitinated mitochondria with the ER could take place in the presence of a mitophagy signal but in the absence of functional autophagy and mitophagy machineries: MEFs deleted for FIP200 and treated with BX-795, the TBK1 inhibitor, still showed that the ubiquitinated mitochondria were encased in ER strands (Figure 7D white arrows, for three separate examples). Longer IVM treatments produced more examples of ER-encased ubiquitinated structures, consistent with the rest of our work showing that the interaction with the ER is a relatively later step after ubiquitination.

DISCUSSION

During ubiquitin-dependent mitophagy, several protein complexes participate in the targeting of damaged mitochondria for degradation: ubiquitination machinery, receptors that recognize damaged mitochondria, and the autophagic machinery creating the double membrane to engulf them (Yoshii and Mizushima, 2015; Yamano et al., 2016). How these machineries are coordinated is unknown. An additional unknown is whether this process depends on a pre-existing membrane, as is the case for the ER during non-selective autophagy, or is it exclusively triggered on the surface of the damaged mitochondria (discussed by Randow and Youle, 2014). In this work, we have provided an integrated view of the sequence of steps involved in making a mitophagosome together with the dynamics of the pathway as seen by live imaging (Figure S5C).

Mechanistically, IVM reduces oxygen consumption in agreement with previous work (Zhu et al., 2017) and fragments mitochondria prior to the induction of mitophagy. Following fragmentation, mitochondria become ubiquitinated, and this was inhibited by inactivation of the DNM1L GTPase that is responsible for mitochondrial fission. Ubiquitination does not involve the PINK1/Parkin proteins but depends on the ubiquitin E3 ligases CIAP1, CIAP2, and TRAF2. These proteins are frequently found in complex (Vince et al., 2008; Zheng et al., 2010) and have been linked to some form of autophagy in the past (Meng et al., 2010; Tang et al., 2013; Hu et al., 2017). In analogy to the function of TRAF2 downstream of celastrol-induced mitophagy (Hu et al., 2017), a plausible pathway for the IVM response is that TRAF2 is activated and ubiquitinates CIAP1 and CIAP2 at the earliest steps, providing the "eat me" signal for mitophagy. Whereas all mitochondria undergo fragmentation very soon after IVM treatment, only the ubiquitin-positive fraction (less than 5% at any given time) become targets for mitophagy. This is similar to PINK1/Parkin-dependent mitophagy: although all mitochondria are covered by overexpressed Parkin, only some become mitophagy substrates at any given time (data not shown). It was technically difficult to determine if the localized loss of mitochondrial potential is a possible signal for ubiquitination.

The ubiquitination step is devoid of any discernible dynamics during live imaging: fragments appear ubiquitin-free and, within 10 s, become ubiquitin positive. Ubiquitinated mitochondrial fragments move in association with ER strands but are not restricted by them until they appear to become entrapped within the strands. This step does not require any of the known downstream machinery such as mitophagy receptors or autophagy proteins.

After ubiquitinated mitochondria are ER restricted, we observed auto-phosphorylation of TBK1 and translocation to the mitochondria-ER site. In MEFs, this occurs before recruitment of the autophagy machinery or of optineurin, the mitophagy receptor. Recent work in PINK1/Parkin-dependent mitophagy also showed TBK1 activation to be an early event, but in that case, optineurin function was required for TBK1 activation in a positive feedback loop (Heo et al., 2015). The importance of TBK1 early in this pathway is also consistent with other work showing co-recruitment of TBK1 with optineurin (Moore and Holzbaur, 2016). The difference of the IVM pathway, which partially uncouples TBK1 from optineurin at the earliest stages, may be that a few molecules of optineurin, not enough to give a fluorescent signal in wide-field conditions but enough to induce the initial TBK1 activation by oligomerization, are involved early. Of note, TBK1 is also important during initial stages of bacterial-targeted autophagy (Thurston et al., 2016).

Downstream of TBK1 activation, we mapped the activity of FIP200 that was genetically uncoupled from ATG13 and ULK1 and ULK2, two other components of the ULK complex. In the absence of FIP200, only P-TBK1 puncta were visible upon IVM induction, but mitophagy adaptors such as optineurin and other autophagy proteins such as WIPI2 did not translocate to the mitophagy sites. In contrast, without ATG13, optineurin-positive puncta still formed. Consistent with this, FIP200 puncta were formed during IVM induction in the ATG13 knockouts. The primacy of FIP200 in the mitophagic response was noted before, and ATG9-positive structures were seen to translocate to "autophagosome formation sites for mitophagy" (Itakura et al., 2012). Based on our finding that ATG9 punctate structures still colocalize with ubiquitin puncta during mitophagy in the absence of FIP200, we suggest that these "autophagosome formation sites for mitophagy" are formed by ubiquitinated mitochondria as they become entrapped by the ER. Activation of the mitophagy receptors such as optineurin was next in our hierarchical analysis, consistent with previous work showing that activated TBK1 phosphorylates optineurin (as well as NDP-52, Tax1BP1, and p62) and causes their translocation during mitophagy

(B) Imaging as in (A) of all frames culminating in complete encasing of the mitochondrial fragment by the ER (arrow in last frame).

(C) Additional example as in (B).

(D) Four-color SIM images of mitochondrial fragments (blue) during IVM-induced mitophagy. ATG13 (green), optineurin (purple), WIPI2 (purple), P-TBK1 (purple), and ER (red) shown as indicated. Extensive panels shown in Figure S5A.

Scale bar, 2 μm.

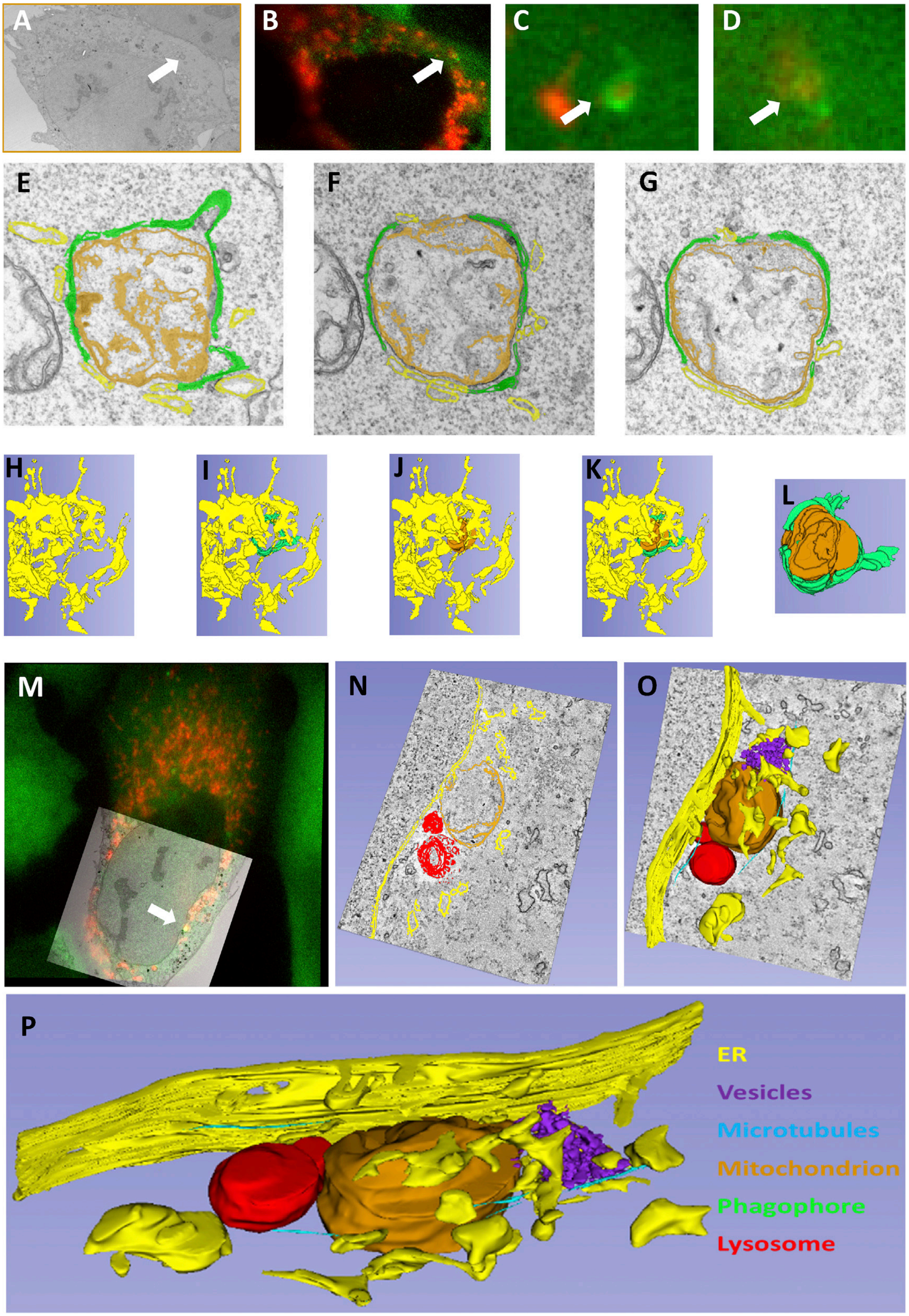

Figure 6. Ultrastructure of the Forming Phagophore during Mitophagy by Correlative Light-Electron Microscopy
Two examples are shown (A–L and M–P) with the color code for all images indicated in (P).
(A) Low-magnification electron micrograph of a cell of interest expressing GFP-ATG13 and dsRED MITO, imaged during IVM treatment.

(legend continued on next page)

(Richter et al., 2016). The position of optineurin upstream of the rest of the autophagy machinery excluding FIP200 and possibly ATG9, is in line with previous work showing sequential translocation first of optineurin and then the omegasome machinery during mitophagy (Wong and Holzbaur, 2014). In our analysis, translocation of ATG13 to the targeted mitochondria was fractionally later than the optineurin translocation, and both optineurin and ATG13 significantly preceded the omegasome step. This is reminiscent of the temporal order of translocation of the ULK and omegasome components to forming autophagosomes during non-selective autophagy (Itakura and Mizushima, 2010; Karanasios et al., 2013). The uncoupling of ATG13 from ULK1 and ULK2, and the non-essential role of the latter in the early steps were surprising, especially since ULK1 translocates to the forming mitophagosome (Lazarou et al., 2015 and data not shown). However, there are now other examples of autophagic processes that separate the function of ATG13 (and FIP200) from ULK1 (Alers et al., 2011; Hieke et al., 2015), and older publications reporting an essential role of the ''ULK complex'' in selective autophagy did not consider the proteins of the complex separately. It is therefore likely that other types of non-selective and selective autophagy may not rely on the ULK1 function for the early step but rather on FIP200 and ATG13 nucleation. Interestingly, even some non-autophagic functions of ATG13 are coupled to FIP200 but uncoupled from ULK1/2 (Kaizuka and Mizushima, 2016).

The dynamics of the engulfment process were extensively studied here, and some were unexpected (Figure S5C). Interaction of optineurin and ATG13 with the targeted mitochondria was not continuous but oscillatory, although for both ATG13 and optineurin, the dynamics of the LC3 structures on the same target were smooth and continuous. The dynamics of the omegasome structures on the targeted mitochondrial fragments were temporally ahead of the LC3 structures and did not exhibit oscillatory behavior. Their movements were more elaborate than the corresponding LC3 movements, and they appeared to ''guide'' where the LC3 signal was deposited. Thus, the PI3P-dependent step in this process is in line with what we have described for non-selective autophagy (Karanasios et al., 2013). The ATG13 and optineurin discontinuous translocations on the forming mitophagosome may indicate that multiple phagophores form during this process, consistent with our live imaging and EM analysis and in line with some previous work (Yoshii et al., 2011). It is also possible that the covering of large structures with a double membrane creates a lag time between translocation of the early components and the lipidation machinery such that early components come on and off giving time to the lipidation machinery to catch up. We are exploring these possibilities by mathematical modeling (Dalle Pezze et al., personal communication). The remarkable outcome of this mechanism is that mitochondrial fragments are precisely engulfed in each mitophagosome with very little empty space evident (our work but see also Sawa-Makarska, and Martens, 2014; Nakatogawa and Ohsumi, 2014).

What ensures such tight fit and efficiency? We have documented using live imaging, super-resolution microscopy, and EM that the ER is intimately associated with the formation of the mitophagosome. Importantly, we did not observe ER strands threading in and out of the autophagic structure—as is the case for non-selective autophagy—but rather, ER strands appeared as a continuation and a cradle for the forming mitophagosomes (Figure S5C). This ER association may supply lipids and provide an anchor point akin to what happens in non-selective autophagy for the ULK and VPS34 complexes to coordinate the translocation events (Ktistakis and Tooze, 2016; Hurley and Young, 2017). The physical proximity of the ubiquitinated mitochondria with the ER (evident even when the rest of the mitophagic machinery is inhibited) may also enable the mitophagy receptors to cluster around their target. Why is this necessary, and why the mitochondrial fragments could not, on their own, provide all the spatial clues necessary for the formation of the mitophagosome? This likely happens for a ''kiss and run'' mechanism whereby the autophagy machinery takes away a piece of damaged mitochondrion leaving the rest intact (Soubannier et al., 2012; Yang and Yang, 2013; Yamashita et al., 2016). For a response such as the one investigated here, the key may be the dynamic behavior of the optineurin and ATG13 components. These proteins do not stay on the targeted mitochondrial fragments continuously, but they come on and off several times during the engulfment process. At the same time, mitochondrial fragments themselves are not stationary but can move long distances around the cell while being engulfed. An ER enclosure may restrict diffusion and allow more efficient targeting of an oscillating machinery to a moving target.

STAR★METHODS

Detailed methods are provided in the online version of this paper and include the following:

- KEY RESOURCES TABLE
- LEAD CONTACT AND MATERIALS AVAILABILITY
- EXPERIMENTAL MODEL AND SUBJECT DETAILS
- METHOD DETAILS
  - Compounds
  - Immunofluorescence Microscopy
  - Super Resolution Microscopy
  - Live Imaging
  - Correlative Light and Electron Microscopy (CLEM)

(B) The corresponding (correlated) fluorescent image.
(C and D) last two frames form live imaging of the cropped region indicated by white arrows in (A) and (B).
(E–G) Images from three sequential serial sections of the same mitophagy event. Green areas indicate phagophore, yellow areas indicate ER-like membranes, and brown membrane is the engulfed mitochondrion.
(H–L) Five views of a 3D model of a mitophagosome with ER alone (H), ER plus phagophores (I), ER plus mitochondria (J), ER plus phagophores plus mitochondria (K) and phagophore plus mitochondria (L); see also Video S7.
(M) Correlation of a low-magnification EM image and the fluorescent image obtained during live imaging.
(N) One 2 nm slice through the tomogram of the event indicated by the white arrow in (M).
(O) Same area as in (N), with a 3D model included.
(P) The segmented model of the event shown in (N) and (O).

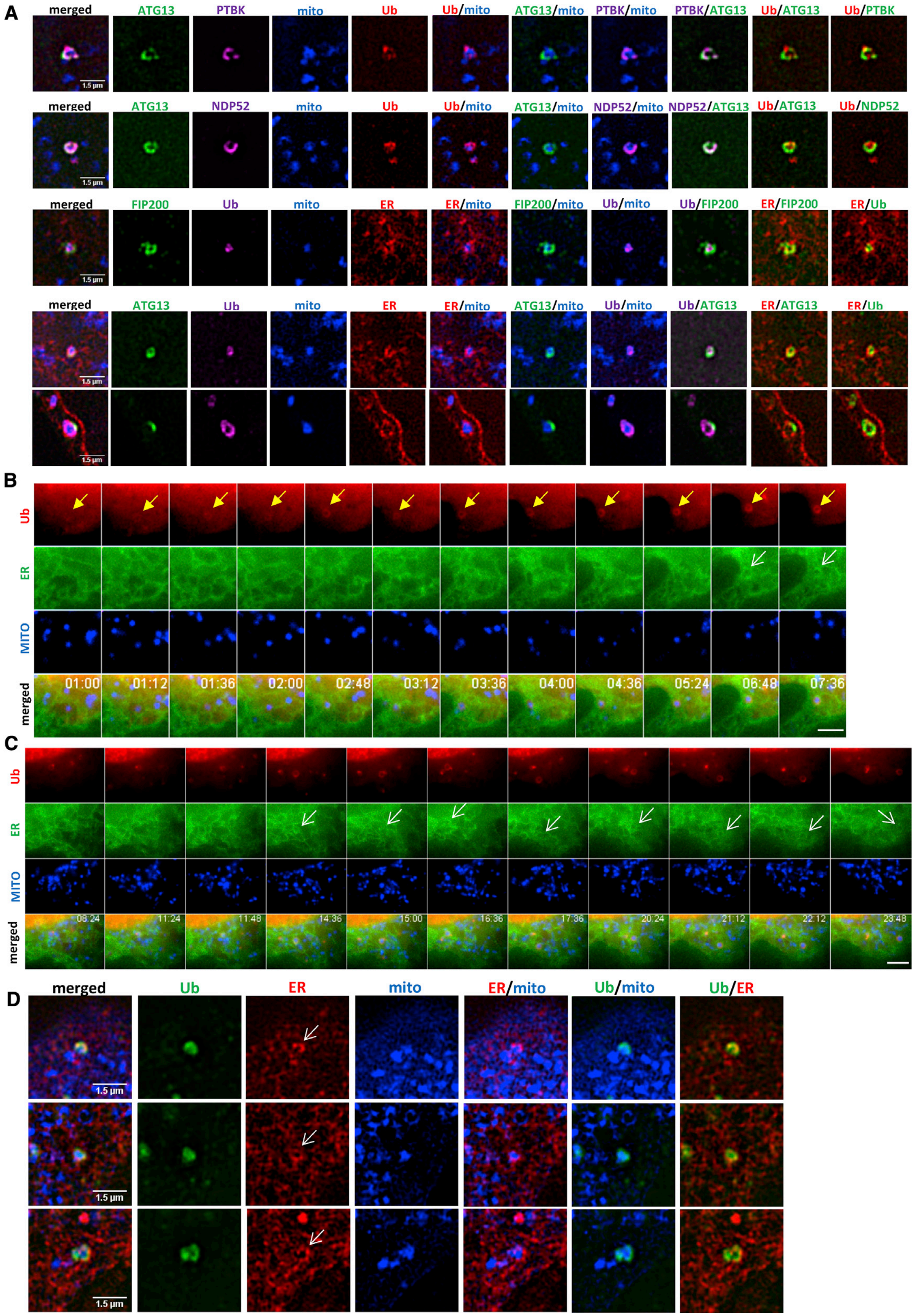

Figure 7. Ubiquitinated Mitochondria Associate with the ER during Mitophagy

(A) Four-color SIM images of mitochondrial fragments (blue) during IVM-induced mitophagy with ATG13 or FIP200 (green), mitophagy adaptors (P-TBK1, NDP-52, and ubiquitin as indicated), and the ER.

(legend continued on next page)

- ○ Lysates and Immunoblots
- ○ Isolation of Ubiquitin-Containing Proteins After IVM Treatment
- ○ siRNA Experiments
- ○ Oxygen Consumption Rate Measurement
- ● QUANTIFICATION AND STATISTICAL ANALYSIS
  - ○ General
  - ○ Note on Live Imaging
- ● DATA AND CODE AVAILABILITY

SUPPLEMENTAL INFORMATION

Supplemental Information can be found online at https://doi.org/10.1016/j.devcel.2019.06.016.

ACKNOWLEDGMENTS

We thank our colleagues Masaaki Komatsu, Noboru Mizushima, and Sharon Tooze for the various ATG KO MEFs. We thank Elise Jacquin for the ATG13 KO HEK293 cells. We thank Professor Sumio Sugano for plasmid pEF321-T and Hanneke Okkenhaug for help in image analysis. Initial characterization of optineurin antibodies in MEF cells was done by Fiorella Cugliandolo. We are grateful and greatly indebted to Dr. David Oxley and Mrs. Judith Webster for carrying out the ubiquitin mass spec work at the Babraham Institute proteomics facility. Our work is supported by the Biotechnology and Biological Sciences Research Council (N.T.K. group) and the Academy of Finland (E.L.E. and S.R.G.). Also supported by COST Action BM1307 Proteostasis.

AUTHOR CONTRIBUTIONS

The project was designed by N.T.K. Work was performed by M.Z., S.R.G., Z.L., M.M., R.S., M.S., J.S., E.K., C.K.-I., H.V., E.J., and S.W. Live imaging and SIM experiments were coordinated and supervised by S.W. E.M. was coordinated and supervised by E.-L.E. The paper was written by N.T.K. with contributions from M.Z., S.R.G., F.B., A.M.S., and E.-L.E. Funding acquisition was by N.T.K., E.-L.E., A.M.S., and F.B.

DECLARATION OF INTERESTS

The authors declare no competing interests.

Received: March 9, 2018
Revised: March 26, 2019
Accepted: June 21, 2019
Published: July 25, 2019

REFERENCES

Alers, S., Löffler, A.S., Paasch, F., Dieterle, A.M., Keppeler, H., Lauber, K., Campbell, D.G., Fehrenbacher, B., Schaller, M., Wesselborg, S., et al. (2011). Atg13 and FIP200 act independently of Ulk1 and Ulk2 in autophagy induction. Autophagy *7*, 1423–1433.

Anding, A.L., and Baehrecke, E.H. (2017). Cleaning house: selective autophagy of organelles. Dev. Cell *41*, 10–22.

Bago, R., Malik, N., Munson, M.J., Prescott, A.R., Davies, P., Sommer, E., Shpiro, N., Ward, R., Cross, D., Ganley, I.G., et al. (2014). Characterization of VPS34-IN1, a selective inhibitor of Vps34, reveals that the phosphatidylinositol 3-phosphate-binding SGK3 protein kinase is a downstream target of class III phosphoinositide 3-kinase. Biochem. J. *463*, 413–427.

Belevich, I., Joensuu, M., Kumar, D., Vihinen, H., and Jokitalo, E. (2016). Microscopy image browser: a platform for segmentation and analysis of multidimensional datasets. PLoS Biol. *14*, e1002340.

Bento, C.F., Renna, M., Ghislat, G., Puri, C., Ashkenazi, A., Vicinanza, M., Menzies, F.M., and Rubinsztein, D.C. (2016). Mammalian autophagy: how does it work? Annu. Rev. Biochem. *85*, 685–713.

Birgisdottir, Å.B., Lamark, T., and Johansen, T. (2013). The LIR motif - crucial for selective autophagy. J. Cell Sci. *126*, 3237–3247.

Campbell, W.C., Burg, R.W., Fisher, M.H., and Dybas, R.A. (1984). The discovery of ivermectin and other avermectins. In ‘‘Pesticide Synthesis Through Rational Approaches’’ Chapter 1, pp 5–20. ACS Symposium Series 255, 5–20.

Cardona, A., Saalfeld, S., Schindelin, J., Arganda-Carreras, I., Preibisch, S., Longair, M., Tomancak, P., Hartenstein, V., and Douglas, R.J. (2012). TrakEM2 software for neural circuit reconstruction. PLoS One *7*, e38011.

Chew, T.S., O’Shea, N.R., Sewell, G.W., Oehlers, S.H., Mulvey, C.M., Crosier, P.S., Godovac-Zimmermann, J., Bloom, S.L., Smith, A.M., and Segal, A.W. (2015). Optineurin deficiency in mice contributes to impaired cytokine secretion and neutrophil recruitment in bacteria-driven colitis. Dis. Models Mech. *8*, 817–829.

Crump, A., and Ōmura, S. (2011). Ivermectin, ’wonder drug’ from Japan: the human use perspective. Proc. Jpn. Acad. Ser. B Phys. Biol. Sci. *87*, 13–28.

Dikic, I. (2017). Proteasomal and autophagic degradation systems. Annu. Rev. Biochem. *86*, 193–224.

Dowdle, W.E., Nyfeler, B., Nagel, J., Elling, R.A., Liu, S., Triantafellow, E., Menon, S., Wang, Z., Honda, A., Pardee, G., et al. (2014). Selective VPS34 inhibitor blocks autophagy and uncovers a role for NCOA4 in ferritin degradation and iron homeostasis in vivo. Nat. Cell Biol. *16*, 1069–1079.

Dunlop, E.A., and Tee, A.R. (2014). mTOR and autophagy: a dynamic relationship governed by nutrients and energy. Semin. Cell Dev. Biol. *36*, 121–129.

Fedorov, A., Beichel, R., Kalpathy-Cramer, J., Finet, J., Fillion-Robin, J.C., Pujol, S., Bauer, C., Jennings, D., Fennessy, F., Sonka, M., et al. (2012). 3D Slicer as an image computing platform for the Quantitative Imaging Network. Magn. Reson. Imaging *30*, 1323–1341.

Feldman, R.I., Wu, J.M., Polokoff, M.A., Kochanny, M.J., Dinter, H., Zhu, D., Biroc, S.L., Alicke, B., Bryant, J., Yuan, S., et al. (2005). Novel small molecule inhibitors of 3-phosphoinositide-dependent kinase-1. J. Biol. Chem. *280*, 19867–19874.

Feng, Y., He, D., Yao, Z., and Klionsky, D.J. (2014). The machinery of macroautophagy. Cell Res. *24*, 24–41.

Georgakopoulos, N.D., Wells, G., and Campanella, M. (2017). The pharmacological regulation of cellular mitophagy. Nat. Chem. Biol. *13*, 136–146.

Heo, J.M., Ordureau, A., Paulo, J.A., Rinehart, J., and Harper, J.W. (2015). The PINK1-Parkin mitochondrial ubiquitylation pathway drives a program of OPTN/NDP52 recruitment and TBK1 activation to promote mitophagy. Mol. Cell *60*, 7–20.

Hieke, N., Löffler, A.S., Kaizuka, T., Berleth, N., Böhler, P., Drießen, S., Stuhldreier, F., Friesen, O., Assani, K., Schmitz, K., et al. (2015). Expression of a ULK1/2 binding-deficient ATG13 variant can partially restore autophagic activity in ATG13-deficient cells. Autophagy *11*, 1471–1483.

Hu, M., Luo, Q., Alitongbieke, G., Chong, S., Xu, C., Xie, L., Chen, X., Zhang, D., Zhou, Y., Wang, Z., et al. (2017). Celastrol-induced Nur77 interaction with TRAF2 alleviates inflammation by promoting mitochondrial ubiquitination and autophagy. Mol. Cell *66*, 141–153.e6.

Hurley, J.H., and Young, L.N. (2017). Mechanisms of autophagy initiation. Annu. Rev. Biochem. *86*, 225–244.

Itakura, E., Kishi-Itakura, C., Koyama-Honda, I., and Mizushima, N. (2012). Structures containing Atg9A and the ULK1 complex independently target

(B) Live imaging of HEK293 cells transiently expressing mCherry-MITO, GFP-Ub, and CFP-ER, treated with IVM. White arrow indicates ER association with the ubiquitin-engulfed mitochondrial fragment (marked with yellow arrows).
(C) Imaging as in (B) for longer time (over 9 min). Scale bars (B and C), 2.5 μm.
(D) Three-color SIM imaging of FIP200 KO MEFs transiently transfected with mCherry-ER and GFP-MITO, pre-treated with BX-795 and then with IVM. Shown are examples of ubiquitinated mitochondrial fragments that coincide with the underlying ER (arrows).

depolarized mitochondria at initial stages of Parkin-mediated mitophagy. J. Cell Sci. *125*, 1488–1499.

Itakura, E., and Mizushima, N. (2010). Characterization of autophagosome formation site by a hierarchical analysis of mammalian Atg proteins. Autophagy *6*, 764–776.

Jacquin, E., Leclerc-Mercier, S., Judon, C., Blanchard, E., Fraitag, S., and Florey, O. (2017). Pharmacological modulators of autophagy activate a parallel noncanonical pathway driving unconventional LC3 lipidation. Autophagy *13*, 854–867.

Johansen, T., and Lamark, T. (2011). Selective autophagy mediated by autophagic adapter proteins. Autophagy *7*, 279–296.

Kaizuka, T., and Mizushima, N. (2016). Atg13 is essential for autophagy and cardiac development in mice. Mol. Cell. Biol. *36*, 585–595.

Kanki, T., Wang, K., Cao, Y., Baba, M., and Klionsky, D.J. (2009). Atg32 is a mitochondrial protein that confers selectivity during mitophagy. Dev. Cell *17*, 98–109.

Karanasios, E., and Ktistakis, N.T. (2015). Live-cell imaging for the assessment of the dynamics of autophagosome formation: focus on early steps. Methods *75*, 54–60.

Karanasios, E., Stapleton, E., Manifava, M., Kaizuka, T., Mizushima, N., Walker, S.A., and Ktistakis, N.T. (2013). Dynamic association of the ULK1 complex with omegasomes during autophagy induction. J. Cell Sci. *126*, 5224–5238.

Karanasios, E., Walker, S.A., Okkenhaug, H., Manifava, M., Hummel, E., Zimmermann, H., Ahmed, Q., Domart, M.C., Collinson, L., and Ktistakis, N.T. (2016). Autophagy initiation by ULK complex assembly on ER tubulovesicular regions marked by ATG9 vesicles. Nat. Commun. *7*, 12420.

Khaminets, A., Behl, C., and Dikic, I. (2016). Ubiquitin-dependent and independent signals in selective autophagy. Trends Cell Biol. *26*, 6–16.

Kremer, J.R., Mastronarde, D.N., and McIntosh, J.R. (1996). Computer visualization of three-dimensional image data using IMOD. J. Struct. Biol. *116*, 71–76.

Ktistakis, N.T., and Tooze, S.A. (2016). Digesting the expanding mechanisms of autophagy. Trends Cell Biol. *26*, 624–635.

Kuma, A., Hatano, M., Matsui, M., Yamamoto, A., Nakaya, H., Yoshimori, T., Ohsumi, Y., Tokuhisa, T., and Mizushima, N. (2004). The role of autophagy during the early neonatal starvation period. Nature *432*, 1032–1036.

Kwon, Y.T., and Ciechanover, A. (2017). The ubiquitin code in the ubiquitin-proteasome system and autophagy. Trends Biochem. Sci. *42*, 873–886.

Lamb, C.A., Yoshimori, T., and Tooze, S.A. (2013). The autophagosome: origins unknown, biogenesis complex. Nat. Rev. Mol. Cell Biol. *14*, 759–774.

Lazarou, M., Sliter, D.A., Kane, L.A., Sarraf, S.A., Wang, C., Burman, J.L., Sideris, D.P., Fogel, A.I., and Youle, R.J. (2015). The ubiquitin kinase PINK1 recruits autophagy receptors to induce mitophagy. Nature *524*, 309–314.

Lemasters, J.J. (2005). Selective mitochondrial autophagy, or mitophagy, as a targeted defense against oxidative stress, mitochondrial dysfunction, and aging. Rejuvenation Res. *8*, 3–5.

Lucocq, J., Manifava, M., Bi, K., Roth, M.G., and Ktistakis, N.T. (2001). Immunolocalisation of phospholipase D1 on tubular vesicular membranes of endocytic and secretory origin. Eur. J. Cell Biol. *80*, 508–520.

Mastronarde, D.N. (2005). Automated electron microscope tomography using robust prediction of specimen movements. J. Struct. Biol. *152*, 36–51.

Meng, N., Wu, L., Gao, J., Zhao, J., Su, L., Su, H., Zhang, S., and Miao, J. (2010). Lipopolysaccharide induces autophagy through BIRC2 in human umbilical vein endothelial cells. J. Cell. Physiol. *225*, 174–179.

Mizushima, N., and Komatsu, M. (2011). Autophagy: renovation of cells and tissues. Cell *147*, 728–741.

Mizushima, N., Yoshimori, T., and Ohsumi, Y. (2011). The role of Atg proteins in autophagosome formation. Annu. Rev. Cell Dev. Biol. *27*, 107–132.

Mochida, K., Oikawa, Y., Kimura, Y., Kirisako, H., Hirano, H., Ohsumi, Y., and Nakatogawa, H. (2015). Receptor-mediated selective autophagy degrades the endoplasmic reticulum and the nucleus. Nature *522*, 359–362.

Mony, V.K., Benjamin, S., and O'Rourke, E.J. (2016). A lysosome-centered view of nutrient homeostasis. Autophagy *12*, 619–631.

Moore, A.S., and Holzbaur, E.L. (2016). Dynamic recruitment and activation of ALS-associated TBK1 with its target optineurin are required for efficient mitophagy. Proc. Natl. Acad. Sci. USA *113*, E3349–E3358.

Motley, A.M., Nuttall, J.M., and Hettema, E.H. (2012). Pex3-anchored Atg36 tags peroxisomes for degradation in Saccharomyces cerevisiae. EMBO J. *31*, 2852–2868.

Nakatogawa, H., and Ohsumi, Y. (2014). Autophagy: close contact keeps out the uninvited. Curr. Biol. *24*, R560–R562.

Narendra, D., Tanaka, A., Suen, D.F., and Youle, R.J. (2008). Parkin is recruited selectively to impaired mitochondria and promotes their autophagy. J. Cell Biol. *183*, 795–803.

Nazarko, T.Y., Ozeki, K., Till, A., Ramakrishnan, G., Lotfi, P., Yan, M., and Subramani, S. (2014). Peroxisomal Atg37 binds Atg30 or palmitoyl-CoA to regulate phagophore formation during pexophagy. J. Cell Biol. *204*, 541–557.

Nguyen, T.N., Padman, B.S., and Lazarou, M. (2016). Deciphering the molecular signals of PINK1/Parkin mitophagy. Trends Cell Biol. *26*, 733–744.

Ohsumi, Y. (2014). Historical landmarks of autophagy research. Cell Res. *24*, 9–23.

Okamoto, K. (2014). Organellophagy: eliminating cellular building blocks via selective autophagy. J. Cell Biol. *205*, 435–445.

Okamoto, K., Kondo-Okamoto, N., and Ohsumi, Y. (2009). Mitochondria-anchored receptor Atg32 mediates degradation of mitochondria via selective autophagy. Dev. Cell *17*, 87–97.

Polson, H.E., de Lartigue, J., Rigden, D.J., Reedijk, M., Urbé, S., Clague, M.J., and Tooze, S.A. (2010). Mammalian Atg18 (WIPI2) localizes to omegasome-anchored phagophores and positively regulates LC3 lipidation. Autophagy *6*, 506–522.

Randow, F., and Youle, R.J. (2014). Self and nonself: how autophagy targets mitochondria and bacteria. Cell Host Microbe *15*, 403–411.

Richter, B., Sliter, D.A., Herhaus, L., Stolz, A., Wang, C., Beli, P., Zaffagnini, G., Wild, P., Martens, S., Wagner, S.A., et al. (2016). Phosphorylation of OPTN by TBK1 enhances its binding to Ub chains and promotes selective autophagy of damaged mitochondria. Proc. Natl. Acad. Sci. USA *113*, 4039–4044.

Roberts, R.F., Tang, M.Y., Fon, E.A., and Durcan, T.M. (2016). Defending the mitochondria: the pathways of mitophagy and mitochondrial-derived vesicles. Int. J. Biochem. Cell Biol. *79*, 427–436.

Rodger, C.E., McWilliams, T.G., and Ganley, I.G. (2018). Mammalian mitophagy - from in vitro molecules to in vivo models. FEBS Journal *285*, 1185–1202.

Rogov, V., Dötsch, V., Johansen, T., and Kirkin, V. (2014). Interactions between autophagy receptors and ubiquitin-like proteins form the molecular basis for selective autophagy. Mol. Cell *53*, 167–178.

Sawa-Makarska, J., and Martens, S. (2014). Excluding the unwanted during autophagy. Cell Cycle *13*, 2313–2314.

Saxton, R.A., and Sabatini, D.M. (2017). mTOR Signaling in growth, metabolism, and disease. Cell *168*, 960–976.

Scott, S.V., Guan, J., Hutchins, M.U., Kim, J., and Klionsky, D.J. (2001). Cvt19 is a receptor for the cytoplasm-to-vacuole targeting pathway. Mol. Cell *7*, 1131–1141.

Soubannier, V., McLelland, G.L., Zunino, R., Braschi, E., Rippstein, P., Fon, E.A., and McBride, H.M. (2012). A vesicular transport pathway shuttles cargo from mitochondria to lysosomes. Curr. Biol. *22*, 135–141.

Stolz, A., Ernst, A., and Dikic, I. (2014). Cargo recognition and trafficking in selective autophagy. Nat. Cell Biol. *16*, 495–501.

Suzuki, K., Kondo, C., Morimoto, M., and Ohsumi, Y. (2010). Selective transport of alpha-mannosidase by autophagic pathways: identification of a novel receptor, Atg34p. J. Biol. Chem. *285*, 30019–30025.

Tang, H.W., Liao, H.M., Peng, W.H., Lin, H.R., Chen, C.H., and Chen, G.C. (2013). Atg9 interacts with dTRAF2/TRAF6 to regulate oxidative stress-induced JNK activation and autophagy induction. Dev. Cell *27*, 489–503.

Thurston, T.L., Boyle, K.B., Allen, M., Ravenhill, B.J., Karpiyevich, M., Bloor, S., Kaul, A., Noad, J., Foeglein, A., Matthews, S.A., et al. (2016).

Recruitment of TBK1 to cytosol-invading Salmonella induces WIPI2-dependent antibacterial autophagy. EMBO J. *35*, 1779–1792.

Tumbarello, D.A., Waxse, B.J., Arden, S.D., Bright, N.A., Kendrick-Jones, J., and Buss, F. (2012). Autophagy receptors link myosin VI to autophagosomes to mediate Tom1-dependent autophagosome maturation and fusion with the lysosome. Nat. Cell Biol. *14*, 1024–1035.

Vince, J.E., Chau, D., Callus, B., Wong, W.W., Hawkins, C.J., Schneider, P., McKinlay, M., Benetatos, C.A., Condon, S.M., Chunduru, S.K., et al. (2008). TWEAK-FN14 signaling induces lysosomal degradation of a cIAP1-TRAF2 complex to sensitize tumor cells to TNFalpha. J. Cell Biol. *182*, 171–184.

Walker, S., Chandra, P., Manifava, M., Axe, E., and Ktistakis, N.T. (2008). Making autophagosomes: localized synthesis of phosphatidylinositol 3-phosphate holds the clue. Autophagy *4*, 1093–1096.

Walker, S.A., Karanasios, E., and Ktistakis, N.T. (2017). Correlative live cell and super resolution imaging of autophagosome formation. Methods Enzymol. *587*, 1–20.

Watanabe, Y., Noda, N.N., Kumeta, H., Suzuki, K., Ohsumi, Y., and Inagaki, F. (2010). Selective transport of alpha-mannosidase by autophagic pathways: structural basis for cargo recognition by Atg19 and Atg34. J. Biol. Chem. *285*, 30026–30033.

Whitworth, A.J., and Pallanck, L.J. (2017). PINK1/Parkin mitophagy and neurodegeneration-what do we really know in vivo? Curr. Opin. Genet. Dev. *44*, 47–53.

Wild, P., Farhan, H., McEwan, D.G., Wagner, S., Rogov, V.V., Brady, N.R., Richter, B., Korac, J., Waidmann, O., Choudhary, C., et al. (2011). Phosphorylation of the autophagy receptor optineurin restricts Salmonella growth. Science *333*, 228–233.

Wilson, M.I., Dooley, H.C., and Tooze, S.A. (2014). WIPI2b and Atg16L1: setting the stage for autophagosome formation. Biochem. Soc. Trans. *42*, 1327–1334.

Wong, P.M., Puente, C., Ganley, I.G., and Jiang, X. (2013). The ULK1 complex: sensing nutrient signals for autophagy activation. Autophagy *9*, 124–137.

Wong, Y.C., and Holzbaur, E.L. (2014). Optineurin is an autophagy receptor for damaged mitochondria in parkin-mediated mitophagy that is disrupted by an ALS-linked mutation. Proc. Natl. Acad. Sci. USA *111*, E4439–E4448.

Yamano, K., Matsuda, N., and Tanaka, K. (2016). The ubiquitin signal and autophagy: an orchestrated dance leading to mitochondrial degradation. EMBO Rep. *17*, 300–316.

Yamashita, S.I., Jin, X., Furukawa, K., Hamasaki, M., Nezu, A., Otera, H., Saigusa, T., Yoshimori, T., Sakai, Y., Mihara, K., et al. (2016). Mitochondrial division occurs concurrently with autophagosome formation but independently of Drp1 during mitophagy. J. Cell Biol. *215*, 649–665.

Yang, J.Y., and Yang, W.Y. (2013). Bit-by-bit autophagic removal of parkin-labelled mitochondria. Nat. Commun. *4*, 2428.

Yang, Y., Kitagaki, J., Dai, R.M., Tsai, Y.C., Lorick, K.L., Ludwig, R.L., Pierre, S.A., Jensen, J.P., Davydov, I.V., Oberoi, P., et al. (2007). Inhibitors of ubiquitin-activating enzyme (E1), a new class of potential cancer therapeutics. Cancer Res. *67*, 9472–9481.

Yoshii, S.R., Kishi, C., Ishihara, N., and Mizushima, N. (2011). Parkin mediates proteasome-dependent protein degradation and rupture of the outer mitochondrial membrane. J. Biol. Chem. *286*, 19630–19640.

Yoshii, S.R., and Mizushima, N. (2015). Autophagy machinery in the context of mammalian mitophagy. Biochim. Biophys. Acta *1853*, 2797–2801.

Yu, L., Chen, Y., and Tooze, S.A. (2018). Autophagy pathway: cellular and molecular mechanisms. Autophagy *14*, 207–215.

Zheng, C., Kabaleeswaran, V., Wang, Y., Cheng, G., and Wu, H. (2010). Crystal structures of the TRAF2: cIAP2 and the TRAF1: TRAF2: cIAP2 complexes: affinity, specificity, and regulation. Mol. Cell *38*, 101–113.

Zhu, M., Li, Y., and Zhou, Z. (2017). Antibiotic ivermectin preferentially targets renal cancer through inducing mitochondrial dysfunction and oxidative damage. Biochem. Biophys. Res. Commun. *492*, 373–378.

STAR★METHODS

KEY RESOURCES TABLE

REAGENT or RESOURCE	SOURCE	IDENTIFIER
Antibodies		
Rabbit anti-OPTN	Cayman Chemicals	100000; RRID: AB_10078198
Mouse anti-WIPI2	Bio-Rad	MCA5780GA; RRID: AB_10845951
Rabbit anti-phospho (S172)TBK1	Cell Signalling	5483
Mouse anti-Ubiquitin FK2	Enzo	BML-PW-8810; RRID: AB_311908
Rabbit anti-FIP200	Protein Tech	17250-1-AP; RRID: AB_10666428
Rabbit anti-ATG9	Cell Signalling	13509
Mouse anti-TOMM20	Abcam	ab56783; RRID: AB_945896
Rabbit anti-ATG13	Sigma	SAB4200100; RRID: AB_10602787
Rabbit anti-LC3	Sigma	L7543; RRID: AB_796155
Rabbit anti-NDP52	GeneTex	GTX115378; RRID: AB_10620266
Rabbit anti-Tax1BP1	Protein Tech	14424-1-AP; RRID: AB_2198921
Rabbit anti-VAPA	Sigma	HPA 009174; RRID: AB_1080549
Rabbit anti-VAPB	Sigma	HPA 013144; RRID: AB_1858717
Mouse anti-EEA1	BD Biosciences	610457; RRID: AB_397830
Mouse anti-LAMP2	Developmental Studies Hybridoma Data Bank	H4B4; RRID: AB_2134755
Mouse anti-TUBULIN	Sigma	T6199; RRID: AB_477583
Mouse anti-cytochrome C	Santa Cruz	sc-7159; RRID: AB_2090474
Rabbit anti- phospho(S351)p62	Dr Masaaki Komatsu	N/A
Rabbit anti-TRAF2	Abcam	ab126758; RRID: AB_11145260
Rabbit anti-CIAP1	Abcam	ab108361; RRID: AB_10862855
Rabbit anti-CIAP2	Protein Tech	24304-1-AP
Mouse anti-TBK1	Santa Cruz	sc-398366
Rabbit anti-(S757)pULK1	Cell Signalling	6888
Mouse anti-OPTN	Santa Cruz	sc-166576; RRID: AB_2156554
Mouse anti-GAPDH	Serotec	4699-9555; RRID: AB_2278713
Agrose beads cross linked to anti-ubiquitin antibody FK2	MBL	D058-8; RRID: AB_843667
Chemicals, Peptides, and Recombinant Proteins		
PP242 hydrate	Sigma-Aldrich	P0037; PubChem SID: 329819988
Ivermectin	Sigma-Aldrich	I8898; CAS: 70288-86-7
PYR-41	Sigma-Aldrich	N2915; CAS: 418805-02-4
Antimycin A from Streptomyces sp.	Sigma-Aldrich	A8674; CAS: 1397-94-0
BX- 795	Tocris	4318; CAS: 702675-74-9
Oligomycin	Tocris	4110; CAS: 579-13-5
CCCP	Tocris	0452; CAS: 555-60-2
Rotenone	Abcam	ab143145
Mdivi-1	Enzo	BML-CM127
VPS34-IN1	Kind gift from Ian Ganley	N/A
Mitot Tracker Deep Red FM	Thermo Fisher (Molecular Probes)	M22426
Mitot Tracker Red CMXRos	Thermo Fisher (Molecular Probes)	M7512
Experimental Models: Cell Lines		
Mouse Embryonic Fibroblasts (MEFs) ATG5 KO	Kind gift from Professor Noboru Mizushima, University of Tokyo, Japan	N/A

(*Continued on next page*)

Continued

REAGENT or RESOURCE	SOURCE	IDENTIFIER
Mouse Embryonic Fibroblasts (MEFs) ATG13 KO	Kind gift from Professor Noboru Mizushima, University of Tokyo	N/A
Mouse Embryonic Fibroblasts (MEFs) ATG3 KO	Kind gift from Professor Masaaki Komatsu, Niigata University, Japan	N/A
Mouse Embryonic Fibroblasts (MEFs)ATG7 KO	Kind gift from Professor Masaaki Komatsu, Niigata University, Japan	N/A
Mouse Embryonic Fibroblasts (MEFs) ULK1/ULK2 KO	Kind gift from Dr Sharon Tooze, Crick Institute, London UK	N/A
Mouse Embryonic Fibroblasts (MEFs) ATG9 KO	Kind gift from Dr Sharon Tooze, Crick Institute, London UK	N/A
Mouse Embryonic Fibroblasts (MEFs) OPTN KO	Chew et al., 2015<br>Tumbarello et al., 2012	
Mouse Embryonic Fibroblasts (MEFs) FIP200	Jun-Lin Guan, University of Cincinnati, USA	N/A
HEK 293 KO ATG13	Kind gift from Dr Elise Jacquin and Dr Oliver Florey, Babraham Institute	N/A
HEK 293 GFP DFCP1	Axe et al, 2008	N/A
HEK 293 GFP ATG13	Karanasios et al., 2013	N/A
Oligonucleotides		
Dharmacon siGENOME siRNA smart pools: TAX1BP1	Dharmacon	# L-016892-00
Dharmacon siGENOME siRNA smart pools: CIAP1	Dharmacon	# L-004390-00
Dharmacon siGENOME siRNA smart pools: CIAP2	Dharmacon	# L-004099-00
Dharmacon siGENOME siRNA smart pools: TRAF2	Dharmacon	# L-005198-00
Dharmacon siGENOME siRNA smart pools: DNML1	Dharmacon	# L-012092-00
Recombinant DNA		
CFP LC3	Kind gift from Tamotsu Yoshimori, Osaka University, Japan	N/A
mCherry-Mito	This work	N/A
pEGFP-C1-Atg13	Addgene	N/A
GFP-Ub	Addgene	N/A
mCherry-Ub	This work	N/A
CFP-Mito	This work	N/A
Software and Algorithms		
Nikon Elements (v4.60) SIM plugin (v2.3)	Nikon	https://www.microscope.healthcare.nikon.com/en_EU/products/software/nis-elements/nis-elements-advanced-research
Olympus cellSens (v1.17)	Olympus	https://www.olympus-lifescience.com/en/software/cellsens/
Seahorse XF24 1.8.1	Agilent	https://www.agilent.com/cs/pubimages/misc/readme-xf24-181.pdf
Prism (Graphpad Software)		https://www.graphpad.com/scientific-software/prism/
Zen2 (blue edition) (Carl Zeiss,file version 2.0.14283.302, Germany)	Carl Zeiss	https://www.zeiss.com/microscopy/int/products/microscope-software/zen-lite.html
ANOVA in XLSTAT	XLSTAT	https://www.xlstat.com/en/
Fiji X 64 (Imaje J)		https://fiji.sc/

LEAD CONTACT AND MATERIALS AVAILABILITY

Further information and requests for reagents may be directed to, and will be fulfilled by, the Lead Author, Dr. Nicholas T. Ktistakis (nicholas.ktistakis@babraham.ac.uk).

EXPERIMENTAL MODEL AND SUBJECT DETAILS

Mouse Embryonic Fibroblasts (MEFs, sex unknown) were generously donated by the following colleagues: ATG5 KO and ATG13 KO, Professor Noboru Mizushima; ATG3 and ATG7 KO, Professor Masaaki Komatsu; ULK1/ULK2 KO and ATG9 KO, Dr Sharon Tooze. The OPTN knockout MEFs were developed from OPTN deficient mice (Chew et al., 2015) as previously described (Tumbarello et al) and immortalized with the SV40 large T-antigen (Kuma et al., 2004). HEK-293 cells (sex, Female) deficient for ATG13 (kindly donated by Dr Elise Jacquin and Dr Oliver Florey) were derived as recently described (Jacquin et al., 2017). HEK-293 cells stably expressing GFP-ATG13 and GFP-DFCP1 have been described before and were isolated after selection in geneticin (Karanasios et al., 2013).

METHOD DETAILS

Compounds

PP242, IVM, PYR-41, and antimycin A were purchased from Sigma-Aldrich (now Merck). BX-795, oligomycin, and CCCP were purchased from Tocris Biosciences. VPS34 INH1 was a kind gift of Dr. Ian Ganley. All compounds were dissolved in DMSO as 1000X stocks and were used at these final concentrations: PP242 1μM; IVM 20 μM (in the original screen it was used at 10 μM); PYR-41 30 μM; BX-795 5 μM, CCCP 4 μM, oligomycin 10 μM, antimycin A 10 μM.

Immunofluorescence Microscopy

Protocols have been extensively described recently (Lucocq et al., 2001; Karanasios, and Ktistakis, 2015). In brief, cells on coverslips were fixed in 3.7% Formaldehyde, permeabilized in 0.1% NP40 and stained in a blocking solution containing fish gelation and 0.05% NP40. Antibodies used and their final dilution are as follows: rabbit anti-OPTN (Cayman Chemicals), 1:100; mouse anti-WIPI2 (Bio-Rad), 1:200; rabbit anti-phospho(S172)TBK1 (Cell Signalling), 1:50; mouse anti-Ubiquitin FK2 (Enzo), 1:100; rabbit anti-FIP200 (ProteinTech), 1:100; rabbit anti-ATG9 (Cell Signalling), 1:100; mouse anti-TOMM20 (Abcam), 1:100; rabbit anti-ATG13 (Sigma), 1:100; rabbit anti-LC3 (Sigma), 1:150; rabbit anti-NDP52 (GeneTex), 1:100; rabbit anti-Tax1BP1 (ProteinTech), 1:100; rabbit anti-VAPA (Sigma), 1:200; rabbit anti-VAPB (Sigma), 1:200; mouse anti-EEA1 (BD Biosciences), 1:70; mouse anti-LAMP2 (Developmental Studies Hybridoma Data Bank), 1:200; mouse anti-TUBULIN (Sigma), 1:300; mouse anti-cytochrome C (Abcam), 1:200; rabbit anti-phospho(S351)p62 (kind gift from Dr Masaaki Komatsu), 1:100; rabbit anti-TRAF2 (Abcam) 1:100.

Super Resolution Microscopy

A Nikon N-SIM system was used, comprising Nikon Ti-E microscope, Nikon 1.49 N.A. objective, Andor iXon 897 EM-CCD camera, Nikon SIM illuminator, Nikon LU5A laser bed and controlled using Nikon Elements software. Raw 3D-SIM images were acquired (15 images representing 5 phases and 3 rotations at each focal plane) typically using 100 ms exposure, 5.1 conversion gain and 150 EM gain. Image stacks were acquired with a 120 nm step interval and reconstructed into super resolved images using the volumetric reconstruction algorithm in the Nikon software. Excitation/emission for the different fluorescent labels was as follows: mTurquoise 405 nm ex, 447/60 em; GFP 488 nm ex, 525/50 em; mCherry 561 nm exn 607/36 em; Alexa Fluor 647 643 nm ex, 692/40 em.

Live Imaging

Two wide-field imaging systems were used to capture images of live cells: a Nikon Ti-E-based system and an Olympus cellSens. Details of our live imaging protocols were described in Karanasios et al (2013); Karanasios et al (2016). Briefly, cells were plated onto 60 mm dishes and transiently transfected with the relevant plasmids. After 24 hr, cells were replated onto 22-mm-diameter glass coverslips and used for imaging on the following morning. Throughout live imaging, cells were maintained at 37°C in a full enclosure incubation system. The Nikon Ti-E-based system comprised a Nikon Ti-E microscope, 100x 1.4 N.A. objective (Nikon), SpecraX LED illuminator (Lumencor), 410/504/582/669-Di01 and Di01-R442/514/561 dichroic mirrors (Semrock), Hamamatsu Flash 4.0 sCMOS camera, emission filter wheel (Sutter Instruments) and was controlled using Nikon Elements software. The Olympus cellSens system comprised of Olympus IX83 microscope, 100x 1.49 N.A. objective (Olympus), pE-4000 LED illuminator (CoolLED), Hamamatsu Flash 4.0 sCMOS camera, ZT440-445/488-491-594 dichroic mirror (Chroma), Olympus filter wheels on excitation and emission paths and was controlled using Olympus cellSens software.

Correlative Light and Electron Microscopy (CLEM)

HEK-293 cells stably expressing GFP-ATG13 were transfected with dsRED MITO for 24 hr and were then replated onto poly lysine-coated, 35 mm gridded MatTek glass bottom dishes to reach 50% confluency on the next day. The cells were treated with 20 μM IVM for 35 min before imaging. After setting the samples on the stage of the Nikon Ti-E microscope at 37°C 5% CO2, they were imaged for an additional 20 min using a 100x 1.4 NA objective and frames were acquired every 10 sec. Before the last imaging frame, 4%

paraformaldehyde in 0.2 M Hepes, pH 7.4, was added to make the final concentrations of 2% paraformaldehyde and 0.1 M M Hepes. After 10 min incubation, the fixative was replaced with 0.2M Hepes buffer to allow capturing a confocal z stack (100x 1.4 NA objective) and the grid co-ordinates were imaged by 10x10 montage bright field imaging (100x 1.4 NA objective). A second round of fixation followed in 2% glutaraldehyde (Sigma EM grade) in 0.2M Hepes (pH 7.4) for 2h. Cells were then osmicated for 1 hr at room temperature with a solution containing 1% OsO4, 0.1M CH32AsO2Na (cacodylate), pH 7.4, 15mg/ml K4[Fe(CN)6. Cells were then sequentially washed with 0.1M cacodylate buffer and water before incubating with 1% uranyl acetate at 4°C for 1 h. After extensive washes in water, samples were dehydrated stepwise in ethanol (50%, 70%, 96%, and 100%). Finally the cells were flat embedded, infiltrated in EPON resin (TAAB 812 ref. T030) for 2 h and baked at +60°C overnight (>14h). The samples were then processed for either electron tomography or conventional thin sectioning. Samples were sectioned at either 60 or 100 nm thickness and picked up on single slot grids. Samples were then stained with 0.5% uranyl acetate for 30 min and 3% lead citrate for 1 min before imaging at 80 kv on a Jeol JEM-1400 (Jeol) equipped with Gatan Orius SC 1000B bottom mounted CCD-camera (Gatan). For tomography, semi thick 230 nm sections were prepared and picked up on single slot grids. 10 nm colloidal gold particles were added to the sections to serve as fiducial markers in the tomogram alignment. Dual axis tilt series were acquired using SerialEM software (Mastronarde, 2005) on a Technai FEG20 microscope (FEI, the Netherlands) at 200 kV with 11500X magnification, over a tilt range of ±62 degrees. Image correlation for correlative light-electron microscopy was done using the TrakEM2 (Cardona et al., 2012) module of Fiji by transforming the fluorescent image and superimposing it on top of a low-magnification EM image of the cell of interest, using mitochondria as the main fiducial markers. Images from the 60 and 100 nm serial sections were aligned using the TrakEM2 module of Fiji, segmented using the Microscope image browser (Belevich et al., 2016) and the three-dimensional models were visualized using 3D Slicer (Fedorov et al., 2012). Tilt series were reconstructed into tomograms and then aligned using IMOD (Kremer et al., 1996), segmented using Microscope image browser and the models visualized using 3D Slicer.

Lysates and Immunoblots

Cells were lysed on ice in 100-140 μL of lysis buffer [50 mM Tris pH 8.0, 50 mM KCl, 1mM EDTA pH 8.0, 1% IGEPAL, 0.6 mM PMSF, Complete Mini, EDTA-free tablet (Roche)] supplemented with 10 % 50mM NaF, 0.1% 10mg/ml leupeptin and 0.5% 0.2M Sodium orthovanadate. Lysates were collected and centrifuged for 10 min at 14,000 rpm at 4°C. The supernatant was collected in fresh tubes on ice. Protein concentration of the lysates was determined using the BCA protein assay kit (Thermo Scientific Pearce). For electrophoresis, samples were combined with 2 x Laemmli sample buffer [20mM Tris-Cl pH 6.8, 2% sodium dodecyl sulphate (SDS), 10% glycerol, 0.1M dithiolthreitol (DTT)] in a 1:1 ratio. Samples were then heated for 90 seconds at 95°C before loading. Gels were wet-transferred overnight to 0.45μm Immobilon-P transfer membranes (Millipore). Incubations with primary and secondary antibodies, and signal development using ECL (ECL Western Blotting Detection Reagent, GE Healthcare/Amersham Biosciences) followed standard protocols. For immunoblots, the following antibodies at the indicated dilutions were used: rabbit anti-ATG9 (Cell Signalling), 1:1500; rabbit anti-ATG13 (Sigma), 1:1000; rabbit anti-FIP200 (ProteinTech), 1:1000; mouse anti-GAPDH (Biogenesis), 1:100,000; mouse anti-OPTN (Santa Cruz), 1:500; rabbit anti-phospho(S172)TBK1 (Cell Signalling), 1:1000; rabbit anti-phospho(S757)ULK1 (Cell Signalling), 1:1000 rabbit anti-TAX1BP1 (homemade), 1:3000; mouse anti-TBK1 (SantaCruz), 1:500, rabbit anti-TRAF2 (Abcam) 1:1000; rabbit anti-CIAP1 (Proteintech); rabbit anti-CIAP2 (Proteintech).

Isolation of Ubiquitin-Containing Proteins After IVM Treatment

HEK-293 cells were treated with 15 μM IVM for 45 min and lysed in 0.3% CHAPS buffer containing 40 mM HEPES-Cl pH 7.4, 120 mM NaCl, 2 mM EDTA, 10 mM pyrophosphate, 10 mM glycerophosphate, 50 mM sodium fluoride, 1.5 mM sodium vanadate and one tablet EDTA-free protease inhibitors per 50 ml. At the same time, agarose beads crosslinked to anti-ubiquitin antibody FK2 (Caltag D058-8) were washed in the same lysis buffer and were added to the cleared lysates for binding at 4°C for 60 min. At the end of incubation, beads were washed 4 times with lysis buffer and once with PBS before mass spectrometry or SDS-PAGE and immunoblotting.

siRNA Experiments

WT and OPTN KO MEFs were seeded in 6-well plates to reach 70%–80% confluency 24 h later. Transfections were performed using TransMessenger transfection reagent kit (Qiagen) with SmartPool siRNA oligos against non-targetting and TAX1BP1 (Dharmacon) for 72 hr.

Downregulation of CIAP1, CIAP2 and TRAF2 simultaneously was done as follows: Cells were plated in the morning and transfected for the first time in the afternoon with 80 pmol of each siRNA SmartPool. The cells were re-transfected two days later with the same amount of siRNA, and examined for the various assays two days later.

Oxygen Consumption Rate Measurement

For oxygen consumption rate (OCR) determination, experiments were carried out using a Seahorse XF24 analyzer (Agilent). HEK-293 cells were plated in a 24 well Seahorse cell culture microplate in 3.5 x 10^4 cells/well density 24hrs prior to the experiment. On the day of the experiment the cells were pre-incubated in DMEM supplemented with 5-mM Glucose, 2mM Glutamine and 5mM HEPES-HCl pH 7.4, for 60 min. IVM was titrated to 1, 5 or 10μM and equivalent volume of DMSO was added in the control samples. All the conditions were in quadruplicates. OCR was measured every 6 min to determine basal respiration, ATP synthase activity and proton leak (using 1 μM Oligomycin), and non-mitochondrial respiration (using 1 μM Rotenone and 2 μM Antimycin). For determination of

percentage OCR inhibition rate compared to the basal, the following equation was used: [1 – [(Last rate measurement before Oligomycin injection – non mitochondrial respiration rate)/(last measurement before IVM/DMSO injection – non mitochondrial respiration rate)]] × 100.

QUANTIFICATION AND STATISTICAL ANALYSIS

General

In a single experiment, 10 images (technical repeats) were selected for each treatment condition and quantified using the ImageJ cell counter plugin. Values were then represented as puncta per cell using GraphPad Prism. Data from biological repeats were combined, log-transformed, and a two-way ANOVA was performed to obtain statistical significance and finally plotted as Mean±SD. All statistical analysis was checked by Dr Anne Segonds-Pichon, the statistician of the Babraham Institute.

Note on Live Imaging

The live imaging data in this manuscript are derived from over 400 videos, and what is shown has been reproducibly observed multiple times by more than one scientist.

DATA AND CODE AVAILABILITY

The published article includes all datasets generated and analyzed during this study.

Developmental Cell, Volume *50*

Supplemental Information

Selective Autophagy of Mitochondria on a Ubiquitin-Endoplasmic-Reticulum Platform

Maria Zachari, Sigurdur R. Gudmundsson, Ziyue Li, Maria Manifava, Ronak Shah, Matthew Smith, James Stronge, Eleftherios Karanasios, Caterina Piunti, Chieko Kishi-Itakura, Helena Vihinen, Eija Jokitalo, Jun-Lin Guan, Folma Buss, Andrew M. Smith, Simon A. Walker, Eeva-Liisa Eskelinen, and Nicholas T. Ktistakis

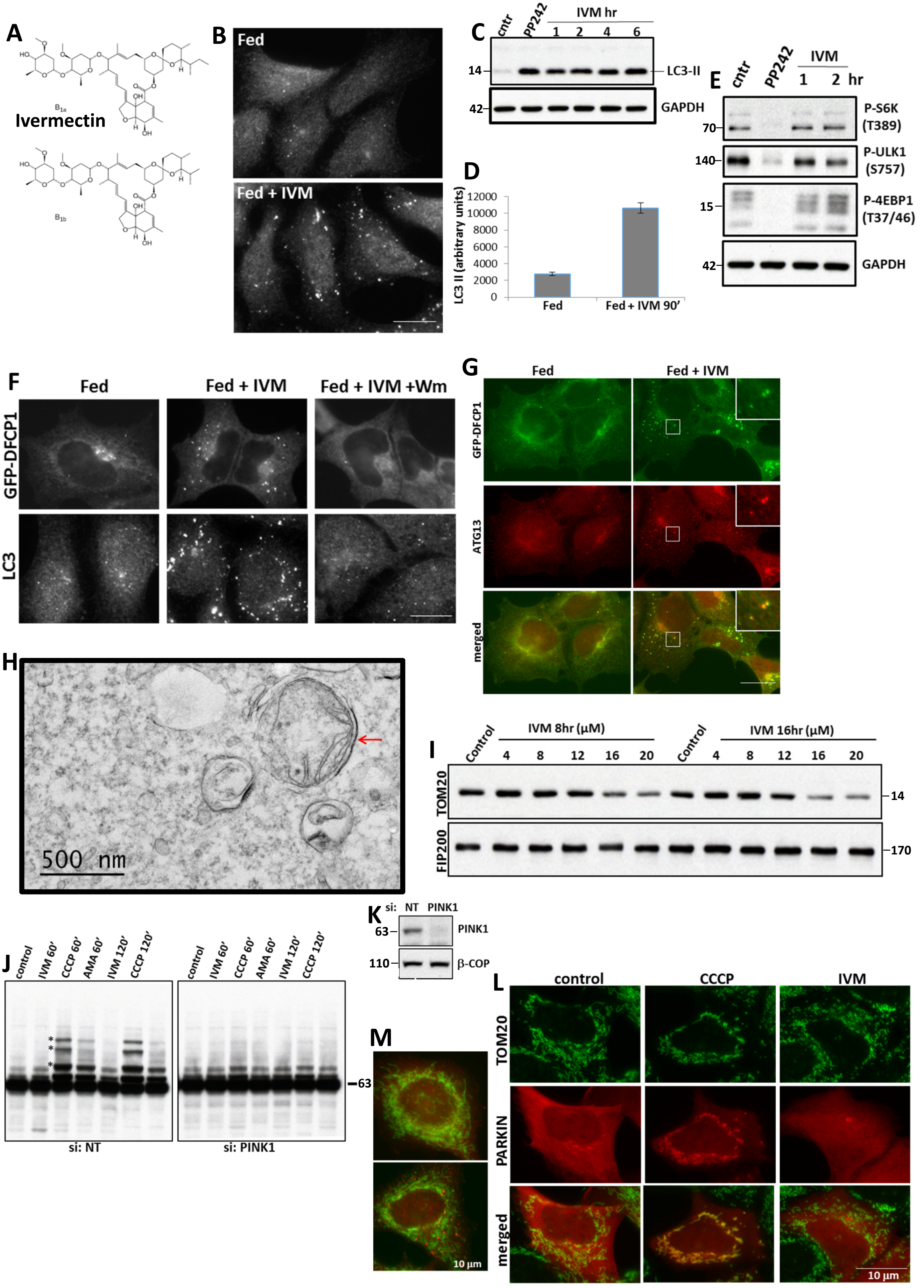

A
Ivermectin
B1a
B1b
B
Fed
Fed + IVM
C
cntr
PP242
IVM hr
1 2 4 6
14
LC3-II
42
GAPDH
D
LC3 II (arbitrary units)
Fed
Fed + IVM 90'
E
cntr
PP242
IVM
1 2 hr
70
P-S6K (T389)
140
P-ULK1 (S757)
15
P-4EBP1 (T37/46)
42
GAPDH
F
Fed
Fed + IVM
Fed + IVM +Wm
GFP-DFCP1
LC3
G
Fed
Fed + IVM
GFP-DFCP1
ATG13
merged
H
500 nm
I
Control
IVM 8hr (μM)
4 8 12 16 20
Control
IVM 16hr (μM)
4 8 12 16 20
TOM20
14
FIP200
170
J
control
IVM 60'
CCCP 60'
AMA 60'
IVM 120'
CCCP 120'
63
si: NT
si: PINK1
K
si: NT PINK1
63
PINK1
110
β-COP
M
10 μm
L
control
CCCP
IVM
TOM20
PARKIN
merged
10 μm

Figure S1 related to Figure 1

IVM induces mitophagy. (A) Structure of IVM. (B-D)) HEKcells were treated with 15 μM IVM for 90 min, and examined for LC3 response by iffl and blots. (E)) HEKcells treated with PP242 for 1 hr or with IVM for 1 and 2 hr were lysed and immunoblotted for phospho-S6K, phopsho-ULK1 or phospho-4EBP1. (F) HEKcells stably expressing GFP-DFCP1 (top panels) or parental HEKcells (bottom panels) were treated with 15 μM IVM +/- 100 nM wortmannin and examined for DFCP1 or LC3. (G) HEKcells stably expressing GFP-DFCP1 were treated with 20 μM IVM for 90 min and immunolabelled for ATG13. (H) HEKcells treated with 15 μM IVM were fixed for EM examination. Arrow marks phagophore engulfing a single mitochondrion. (I) HEKcells were treated with the indicated amounts of IVM for 8 hr or 16 hr and then immunoblotted for TOM20 and FIP200 . (J-K)HEKcells were transfected with siRNAs against PINK1 or with non-targeting control (NT) and treated with IVM, CCCP or antimycin A before immunoblotting for MITOFUSIN 2 (J) or PINK1 (K). Asterisks indicate higher mobility forms of MITOFUSIN 2. (L) HEKcells were transiently transfected with mCherry-Parkin and treated for 90 min with CCCP or with IVM as indicated beforeTOM20 immunolabellimg (M) HeLa cells were treated with IVM for 90 min before immunolabelling with WIPI2 and TOM20.

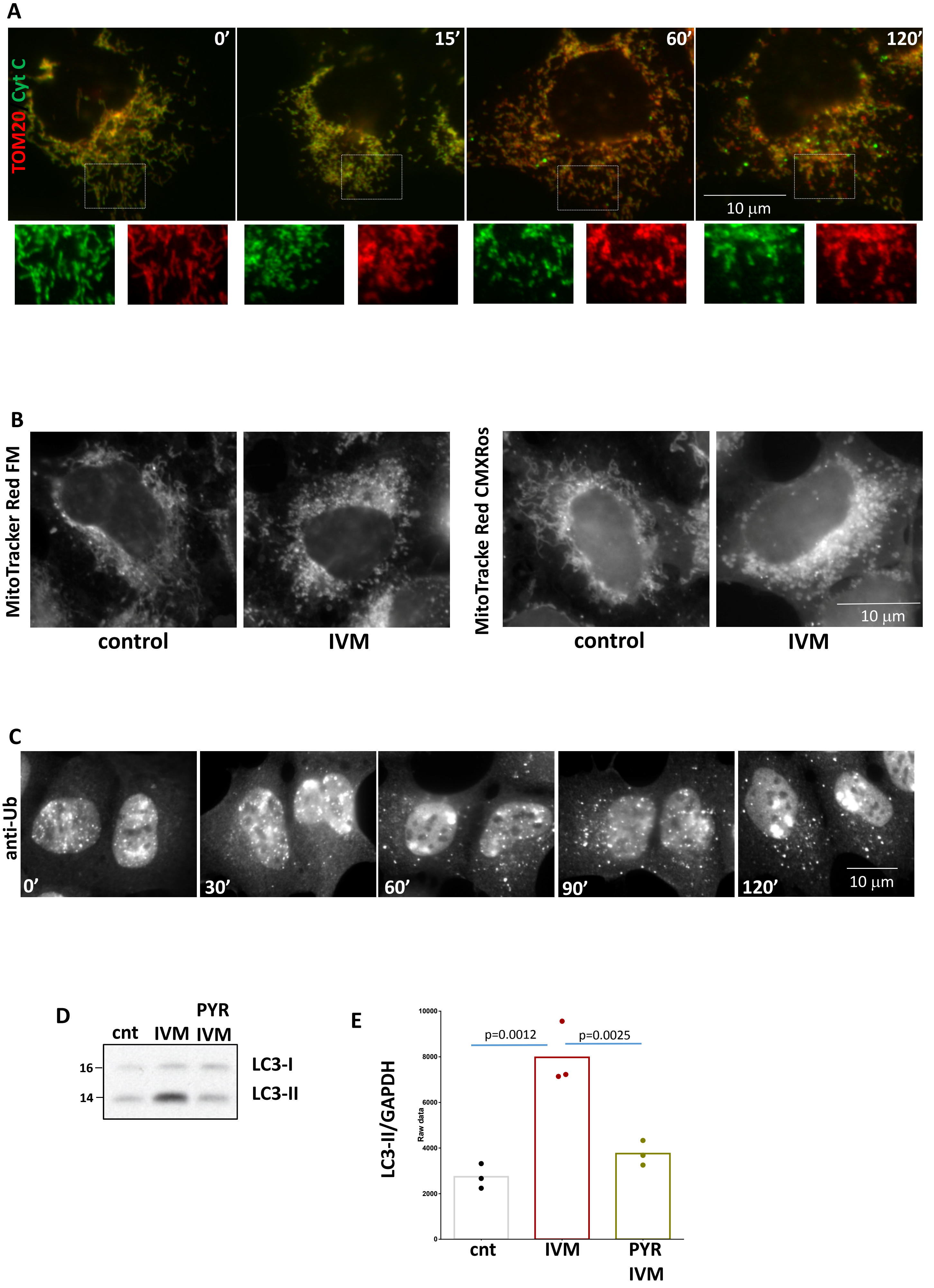
A
TOM20 Cyt C
0’
15’
60’
120’
10 µm
B
MitoTracker Red FM
MitoTracke Red CMXRos
control
IVM
control
IVM
10 µm
C
anti-Ub
0’
30’
60’
90’
120’
10 µm
D
cnt
IVM
PYR
IVM
16
14
LC3-I
LC3-II
E
p=0.0012
p=0.0025
LC3-II/GAPDH
Raw data
10000
8000
6000
4000
2000
0
cnt
IVM
PYR
IVM

Figure S2 related to Figure 1.

IVM does not induce mitochondrial permeabilization; IVM—induced mitophagy depends on ubiquitination. (A) HEK-293 cells were treated with 15 μM IVM for the times indicated and immunolabelled for TOM20 and cytochrome C as indicated. (B) HEK-293 cells were incubated with 50 ng/ml of Mitotracker Red FM or Mitotracker Red CMXRos as indicated for 10 min followed by addition of IVM for an additional 60 min. (C) Cells treated as in A were immunolabelled with antibodies to ubiquitin. (D) HEK-293 cells were treated with IVM in the presence or absence of PYR-41 and lysates were immunoblotted for LC3. (E) Triplicate experiment as shown in D is quantitated.

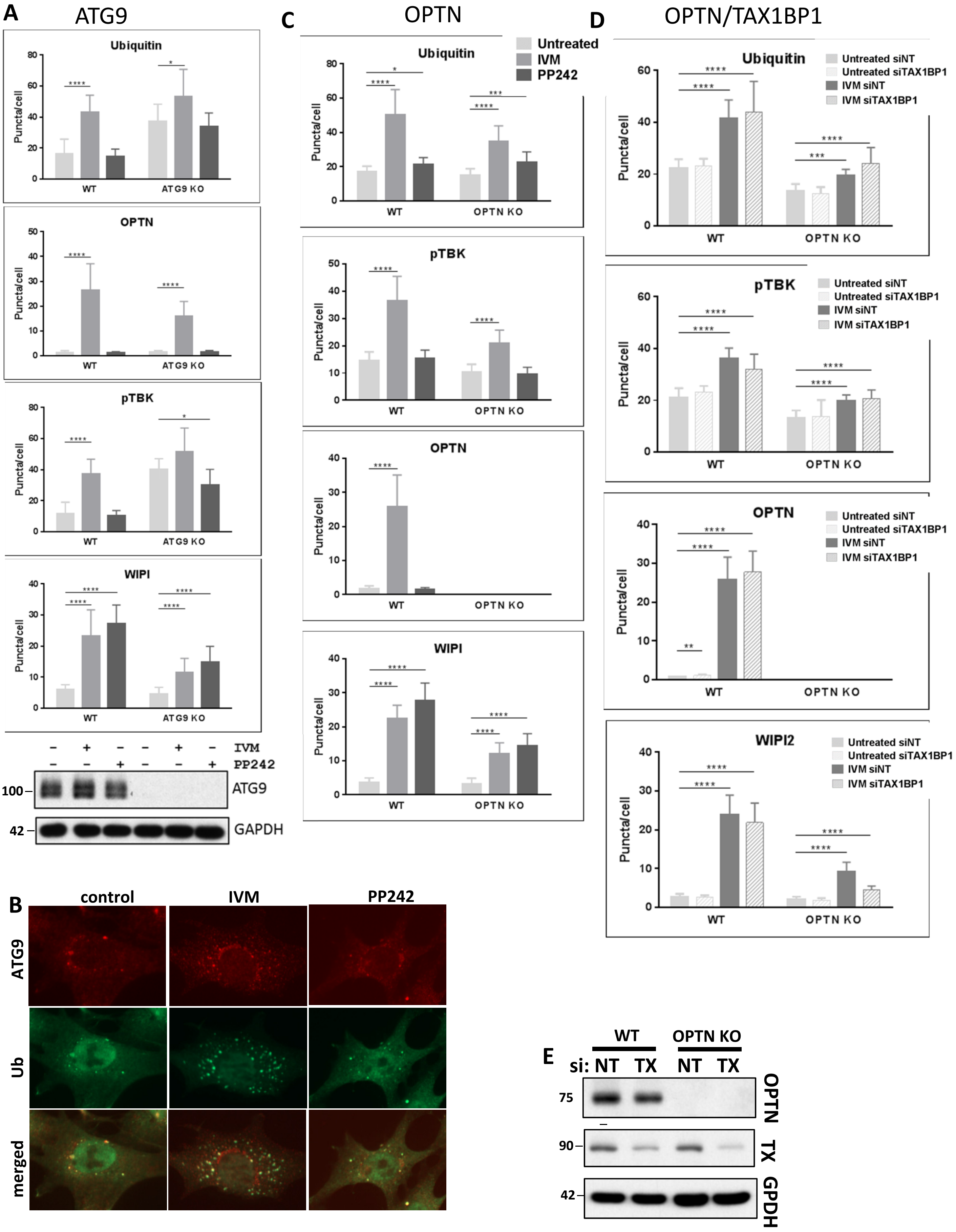

A
ATG9
Ubiquitin
OPTN
pTBK
WIPI
Puncta/cell
WT
ATG9 KO
IVM
PP242
100
42
ATG9
GAPDH
B
control
IVM
PP242
ATG9
Ub
merged
C
OPTN
Untreated
IVM
PP242
OPTN KO
D
OPTN/TAX1BP1
Untreated siNT
Untreated siTAX1BP1
IVM siNT
IVM siTAX1BP1
WIPI2
E
WT
OPTN KO
si: NT TX NT TX
75
90
42
OPTN
TX
GPDH

Figure S3 related to Figures 2-3

Requirement for ATG9, Optineurin and Tax1bp in IVM-induced mitophagy. (A) Wild type MEF cells or ATG9 KOs were treated for 90 min with 15 mM IVM or with 1 mM PP242 and immunolabelled for ubiquitin, phospho-TBK1, Optineurin and WIPI2. Number of puncta for each condition are shown in the graphs. (B) MEF cells deleted for FIP200 were treated for 90 min with 15 mM IVM or with 1 mM PP242 and immunolabelled for ubiquitin and ATG9. (C) Wild type MEF cells or optineurin KOs were treated for 90 min with 15 μM IVM or with 1 μM PP242 and immunolabelled for ubiquitin, phospho-TBK1, Optineurin and WIPI2. Number of puncta for each condition is shown in the graphs. (D) Wild type and Optineurin KO MEFs as in A were transfected with siRNAs against Tax1bp or with a non-targeting oligonucleotide for 72 hrs. The cells were then treated with 15 μM IVM and were immunolabelled with ubiquitin, phospho-TBK1, optineurin and WIPI2. The number of puncta for each condition is shown in the graphs. (E) Lysates from wild type or Optineurin KO MEFs transfected with siRNAs to Tax1bp or with a non-targeting control (NT) and immunoblotted for the indicated proteins.

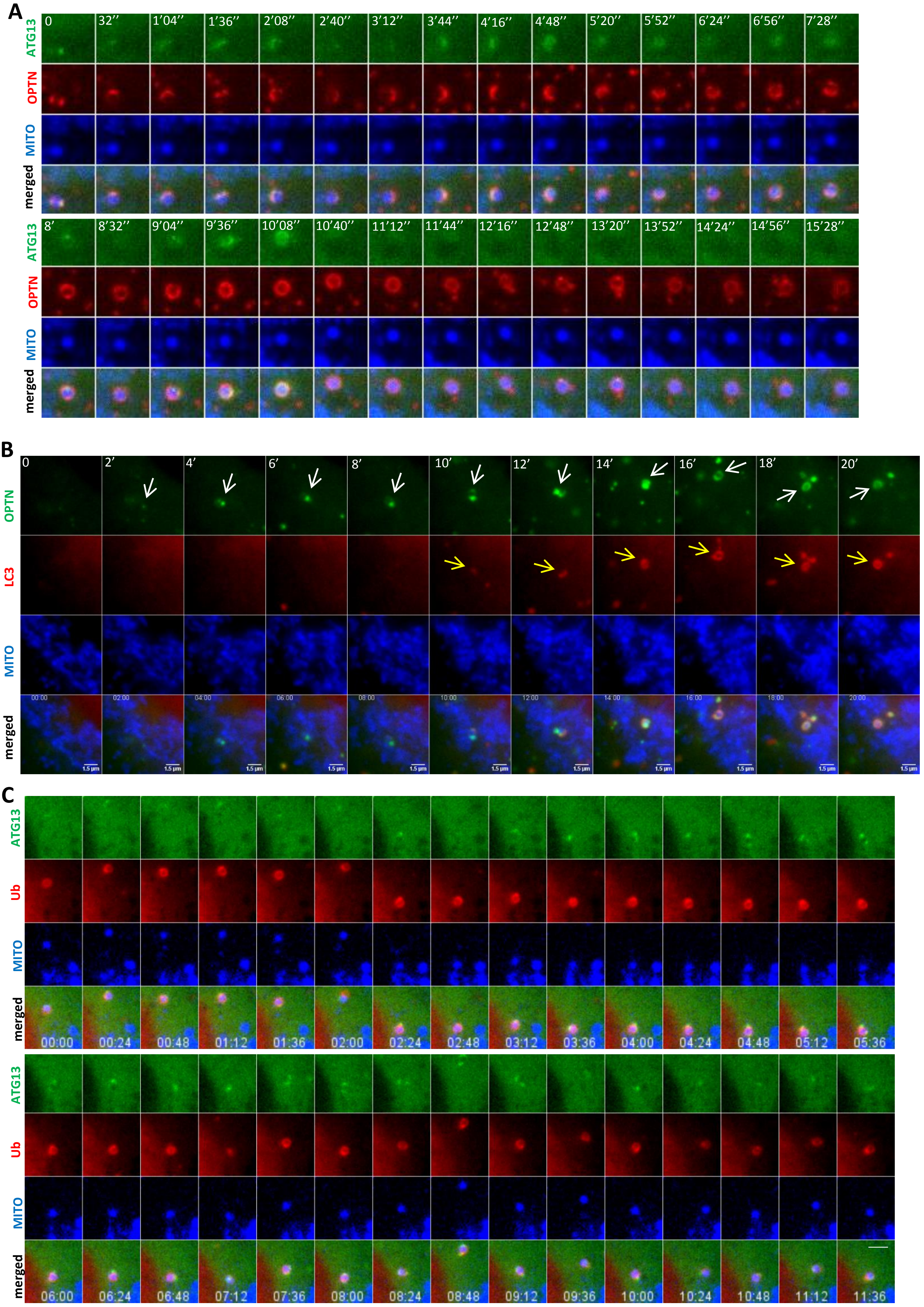

A
ATG13
OPTN
MITO
merged
0
32''
1'04''
1'36''
2'08''
2'40''
3'12''
3'44''
4'16''
4'48''
5'20''
5'52''
6'24''
6'56''
7'28''
8'
8'32''
9'04''
9'36''
10'08''
10'40''
11'12''
11'44''
12'16''
12'48''
13'20''
13'52''
14'24''
14'56''
15'28''
B
OPTN
LC3
MITO
merged
0
2'
4'
6'
8'
10'
12'
14'
16'
18'
20'
C
ATG13
Ub
MITO
merged
00:00
00:24
00:48
01:12
01:36
02:00
02:24
02:48
03:12
03:36
04:00
04:24
04:48
05:12
05:36
06:00
06:24
06:48
07:12
07:36
08:00
08:24
08:48
09:12
09:36
10:00
10:24
10:48
11:12
11:36

Figure S4 related to Figure 4.

Dynamics of optineurin and ubiquitin during mitophagy. (A) Wide field live-imaging of HEK-293 cells stably expressing GFP-ATG13 and transiently transfected with mCherry-optineurin and CFP-MITO (A) or transiently transfected with GFP-optineurin, CFP-LC3 and mCherry-MITO (B) orstably expressing GFP-ATG13 and transiently transfected with mCherry-ubiquitin and CFP-MITO (C). IVM was added for 10 min and then cells were imaged for an additional 60 min. The bar represents 1 μm.

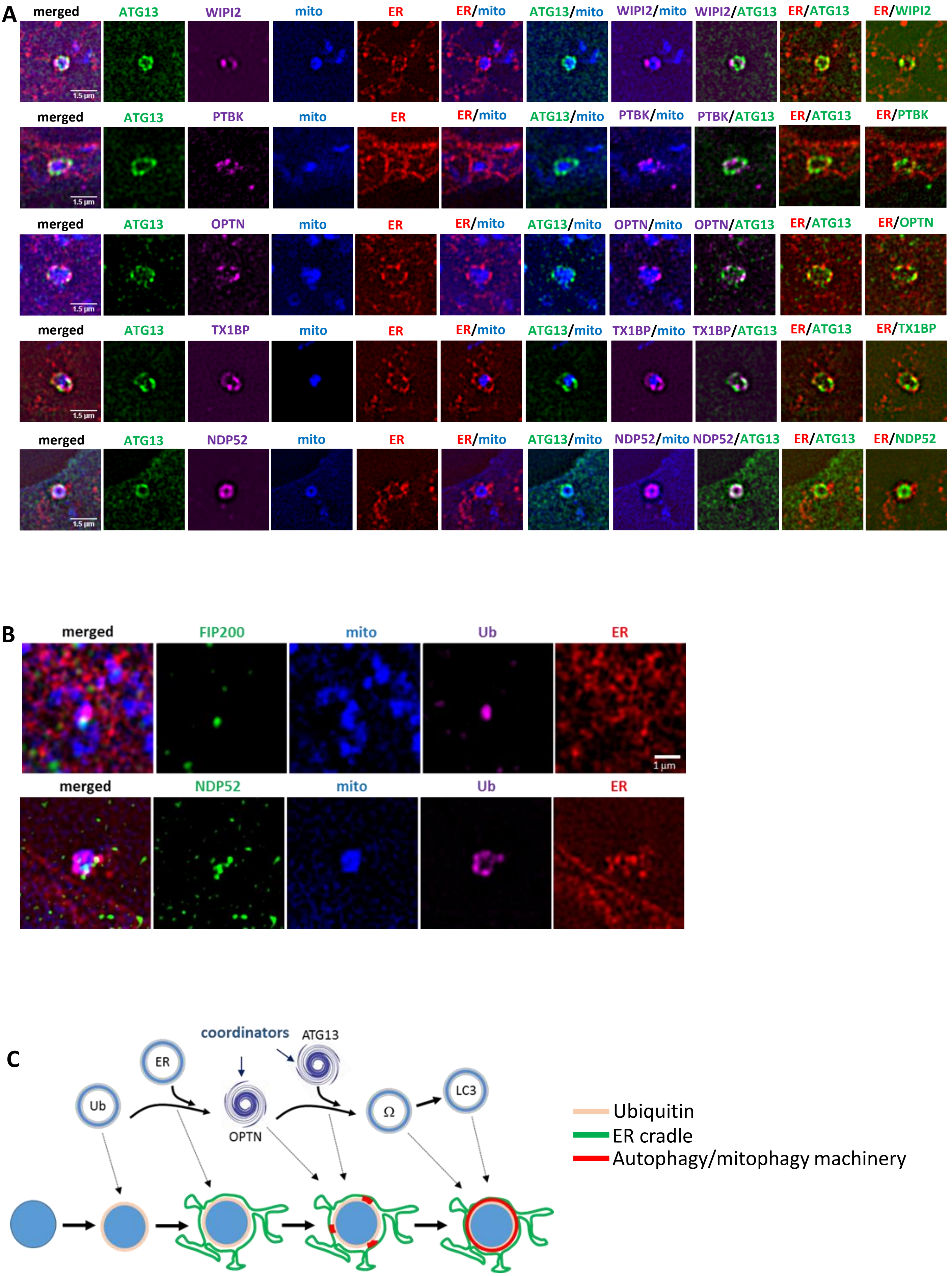
A
merged
ATG13
WIPI2
mito
ER
ER/mito
ATG13/mito
WIPI2/mito
WIPI2/ATG13
ER/ATG13
ER/WIPI2
1.5 µm
merged
ATG13
PTBK
mito
ER
ER/mito
ATG13/mito
PTBK/mito
PTBK/ATG13
ER/ATG13
ER/PTBK
1.5 µm
merged
ATG13
OPTN
mito
ER
ER/mito
ATG13/mito
OPTN/mito
OPTN/ATG13
ER/ATG13
ER/OPTN
1.5 µm
merged
ATG13
TX1BP
mito
ER
ER/mito
ATG13/mito
TX1BP/mito
TX1BP/ATG13
ER/ATG13
ER/TX1BP
1.5 µm
merged
ATG13
NDP52
mito
ER
ER/mito
ATG13/mito
NDP52/mito
NDP52/ATG13
ER/ATG13
ER/NDP52
1.5 µm
B
merged
FIP200
mito
Ub
ER
1 µm
merged
NDP52
mito
Ub
ER
C
coordinators
ATG13
ER
Ub
OPTN
Ω
LC3
Ubiquitin
ER cradle
Autophagy/mitophagy machinery

Figure S5 related to Figure 5.

(A) An expanded set of the images shown in Figure 8 D. Four-color SIM images of mitochondrial fragments (blue) during IVM-induced mitophagy and associated with ATG13 (green), optineurin (purple), WIPI2 (purple), phospho-TBK1 (purple) Tax1BP (purple), NDP-52 (purple) and ER (red) as indicated. Shown are the four individual channels, the four-color overlay and two-color overlays of the various components as indicated. (B) Four-color SIM images of mitochondrial fragments (blue) during IVM-induced mitophagy in HEK-293 cells lacking ATG13. Staining for ER (red) and other components is as indicated. (C) Dynamics of mitophagy as revealed in this work. Several components are involved in the engulfment of the mitochondrial target (top diagram): some cover it smoothly (ubiquitin, ER, omegasomes, LC3, continuous lines) whereas others appear to oscillate on and off and translocate to multiple sites on the target (optineurin and ATG13 shown as an example, swirling lines). We imagine that these components co-ordinate the translocation of the rest of the machinery, including the lipidation complex and LC3. One way to explain these dynamics is that the ubiquitination step leads to some stable interaction of the mitochondrial target with an ER cradle, which provides the spatial clues for organizing the translocation of the autophagy/mitophagy machinery (bottom diagram).

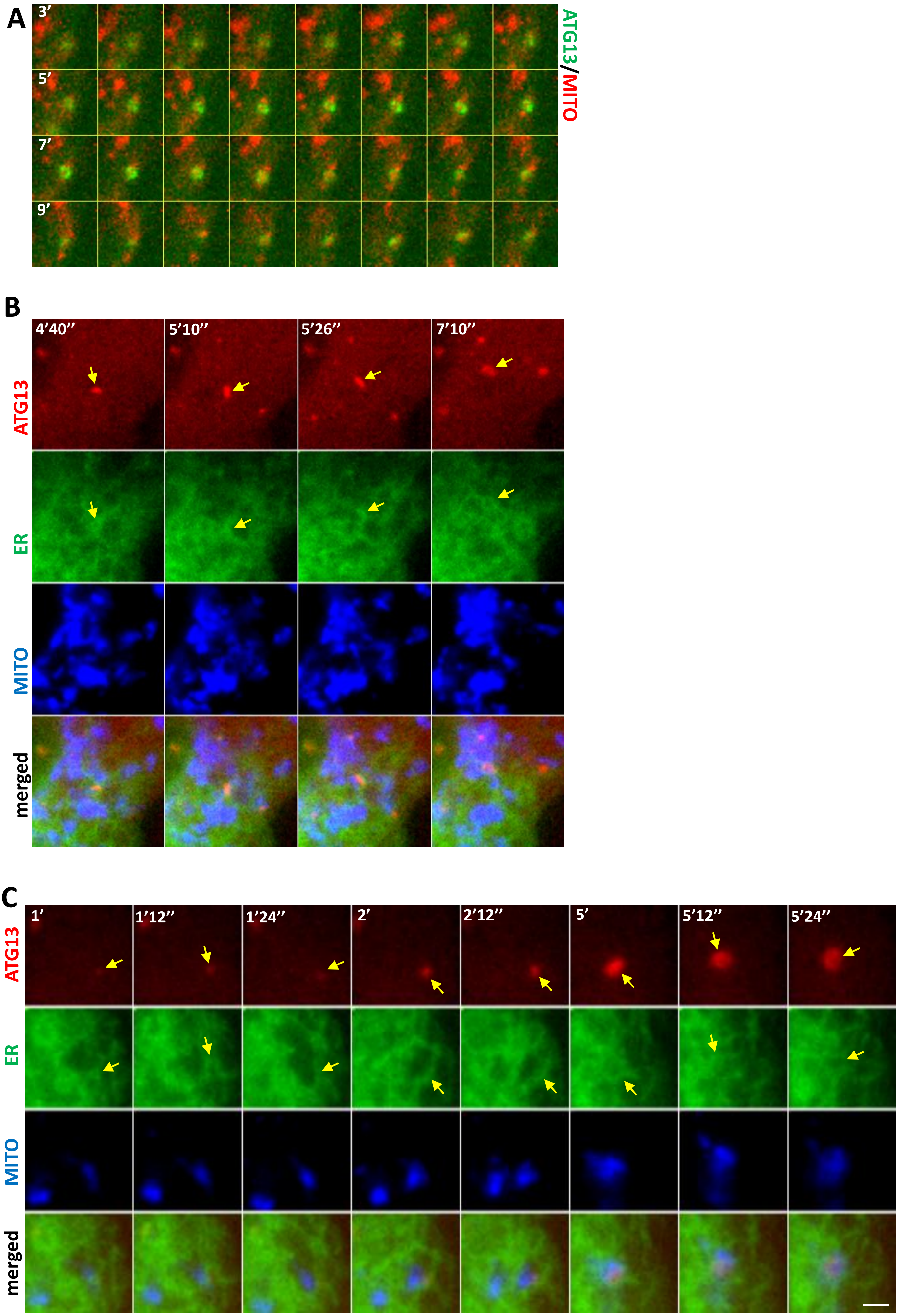
A
3'
5'
7'
9'
ATG13/MITO
B
4'40''
5'10''
5'26''
7'10''
ATG13
ER
MITO
merged
C
1'
1'12''
1'24''
2'
2'12''
5'
5'12''
5'24''
ATG13
ER
MITO
merged

Figure S6 related to Figures 4-5.

Dynamics of mitophagy induced by Antimycin A/Oligomycin (A/O) treatment. (A) HEK-293 cells expressing GFP-ATG13 and mCherry-MITO were treated with A/O for 8 h and then imaged every 12 sec.. (B-C) HEK-293 cells expressing GFP-ATG13, mCherry-MITO and CFP-ER were treated with A/O for 8 h (C) or 18 (D) h and imaged every 12 sec as shown in the selected panels. Arrows indicate the position of ATG13 and ER structures during the mitochondrial engulfment process. The bar in all panels represents 1 μm.

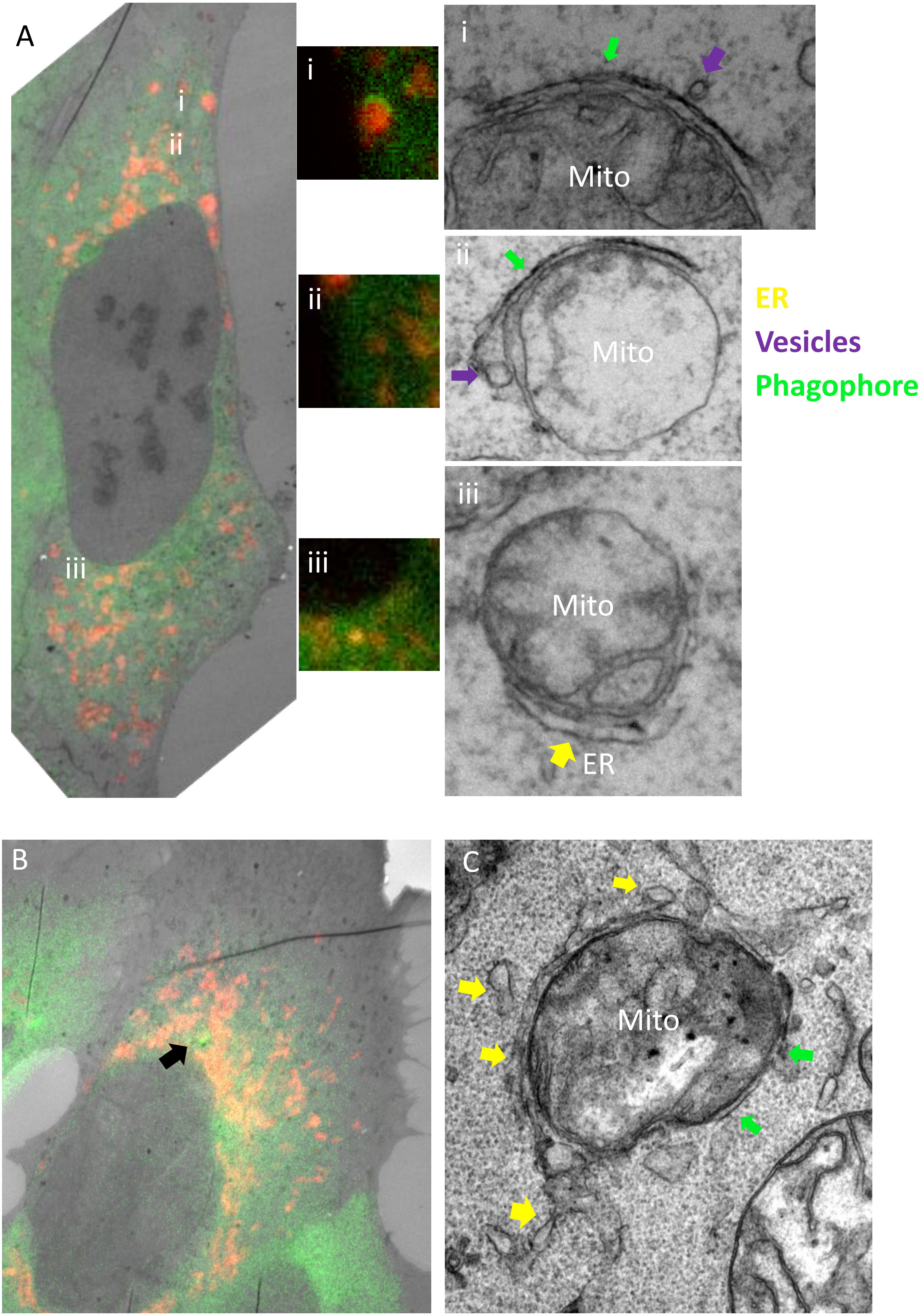
A
i
ii
iii
i
ii
iii
i
Mito
ii
Mito
iii
Mito
ER
ER
Vesicles
Phagophore
B
C
Mito

Figure S7 related to Figure 6.

Correlative light-electron microscopy images of IVM-induced mitophagy. (A) A cell with 3 simultaneous mitophagosomes (i, ii, iii). Events i and ii show a forming phagophore tightly next to the mitochondrion while event iii shows an ER-like structure next to the mitochondrion, although an ATG13 signal is evident in live imaging in all three events. The absence of a phagophore when ATG13 is seen to translocate to the mitochondrion is reminiscent of the event shown in Fig 9 M-P and probably represents the earliest intermediates in mitophagy pathway. (B) A mitophagy event showing how ER (yellow arrows) and phagophore (green arrows) wrap around the targeted mitochondrial fragment.
